# Chromosomal integration of aldo-keto-reductase and short-chain dehydrogenase/reductase genes in *Clostridium beijerinckii* NCIMB 8052 enhanced tolerance to lignocellulose-derived microbial inhibitory compounds

**DOI:** 10.1038/s41598-019-44061-1

**Published:** 2019-05-21

**Authors:** Christopher Chukwudi Okonkwo, Victor Ujor, Thaddeus Chukwuemeka Ezeji

**Affiliations:** 1 Department of Animal Sciences, The Ohio State University, and Ohio State Agricultural Research and Development Center (OARDC), 305 Gerlaugh Hall, 1680 Madison Avenue, Wooster, OH 44691 USA; 2 Bioenergy and Biological Waste Management Program, Agricultural Technical Institute, The Ohio State University, 1328 Dover Road, Wooster, OH 44691 USA

**Keywords:** Metabolic engineering, Metabolic engineering

## Abstract

*In situ* detoxification of lignocellulose-derived microbial inhibitory compounds is an economical strategy for the fermentation of lignocellulose-derived sugars to fuels and chemicals. In this study, we investigated homologous integration and constitutive expression of *Cbei_*3974 and *Cbei_*3904, which encode aldo-keto reductase and previously annotated short chain dehydrogenase/reductase, respectively, in *Clostridium beijerinckii* NCIMB 8052 (*Cb*), resulting in two strains: *Cb*_3974 and *Cb*_3904. Expression of *Cbei_*3974 led to 2-fold increase in furfural detoxification relative to *Cb*_3904 and *Cb*_wild type. Correspondingly, butanol production was up to 1.2-fold greater in furfural-challenged cultures of *Cb*_3974 relative to *Cb*_3904 and *Cb*_wild type. With 4-hydroxybezaldehyde and syringaldehyde supplementation, *Cb*_3974 showed up to 2.4-fold increase in butanol concentration when compared to *Cb*_3904 and *Cb*_wild type. Syringic and vanillic acids were considerably less deleterious to all three strains of *Cb* tested. Overall, *Cb_*3974 showed greater tolerance to furfural, 4-hydroxybezaldehyde, and syringaldehyde with improved capacity for butanol production. Hence, development of *Cb*_3974 represents a significant progress towards engineering solventogenic *Clostridium* species that are tolerant to lignocellulosic biomass hydrolysates as substrates for ABE fermentation.

## Introduction

Renewable feedstocks such as lignocellulosic biomass (LB) and organic municipal wastes are sources of cheap sugars with potential to lower the overall cost of fuels and chemicals production. For instance, large-scale bio-butanol production is currently not economically viable, in part, due to the higher cost of traditional feedstocks such as corn and sugarcane. Solventogenic *Clostridium* species are strict anaerobes capable of converting a wide range of substrates including the major LB-derived sugars—namely, glucose, xylose and arabinose—to acetone, butanol and ethanol (ABE) during ABE fermentation^1^. LB, which is composed of cellulose, hemicellulose and lignin is recalcitrant to mild biochemical deconstruction, hence, requires a pretreatment process to render it amenable to enzymatic hydrolysis. However, while LB pretreatment releases fermentable sugars, it also generates lignocellulose-derived microbial inhibitory compounds (LDMICs) that are deleterious to fermenting microorganisms.

The LDMICs generated during LB pretreatment and hydrolysis include furfural, 5-hydroxymethyl furfural (HMF) and a collection of lignin-derived phenolic compounds^2^. These inhibitors significantly affect microbial growth and metabolism by damaging membranes, inhibiting enzymes, and damaging DNA, in addition to disrupting cellular redox balance, often with concomitant decreases in cellular ATP levels^3–5^. Consequently, LB-derived inhibitors impede industrial-scale utilization of LB-derived sugars as substrates in large-scale fermentation. Considerable research efforts have pursued development of strategies and techniques for inhibitor removal prior to fermentation. These techniques include the use of chemical additives such as dithionite, dithiothreitol, sulfite and calcium hydroxide (over-liming), enzymatic treatments with laccases and peroxidases, liquid-liquid extraction with ethyl acetate or trialkyl amine, liquid-solid extraction with activated carbon or ion exchange resins for inhibitor removal^6–15^. Although effective, these techniques introduce additional detoxification steps, with the attendant increase in overall cost, which diminishes the economic competitiveness of ABE fermentation for bio-butanol production. Additionally, a considerable percentage of fermentable sugars is lost during inhibitor removal, which further affects the economics of the overall process. A cheap and economical strategy for improving large-scale microbial fermentation of LB-derived sugars to fuels and chemicals is to metabolically fortify fermenting microbes with the genetic repertoire to detoxify LB-derived inhibitors *in situ* during fermentation. Towards achieving this goal, our group has focused on identifying genes whose protein products are central to cellular detoxification of LB-derived inhibitors during ABE fermentation^1^. An extensive study of genome-wide transcriptional response of *Clostridium beijerinckii* NCIMB 8052 (hereafter referred to as *Cb*) to furfural stress during ABE fermentation revealed that, of the 721 genes that were differentially expressed, aldo/keto reductase (AKR; *Cbei_3974*) and short-chain dehydrogenase/reductase (*SDR*; *Cbei_*3904) were among the most strongly upregulated genes^16^. This, coupled with the annotated functions of both genes suggest that they likely play a critical role in LDMICs detoxification by *Cb*.

AKR and SDR are NADPH-dependent oxidoreductases that participate in redox reactions that utilize aldehydes (such as furfural and HMF) as substrates. To establish and delineate the roles of the upregulated *AKR* and *SDR* genes in furfural-challenged *Cb*, we cloned (in *Escherichia coli* Rosetta-gami™), overexpressed, purified and characterized the protein products of both genes^1^. Our results showed that the enzyme encoded by each gene (*Cbei_*3974 and *Cbei_3904*) convert furfural to the less toxic furfuryl alcohol using NADPH as cofactor^1^. Furthermore, both enzymes were found to be active on HMF and the phenolic compound, benzaldehyde, which is also co-generated during LB pretreatment. Based on the above findings, we hypothesized that overexpression of *Cbei_3974* and *Cbei_3904* in *Cb* would likely expedite inhibitor detoxification, hence; increase the ability of the resulting strains to tolerate higher concentrations of furanic aldehydes. Such increase in furanic aldehyde tolerance would ultimately enhance solvent production—particularly, butanol—during ABE fermentation in furanic aldehyde-challenged cultures. Whereas initial attempts to clone and express both genes in *Cb* were successful, the combined effect of antibiotic (erythromycin) as a selectable marker for maintaining the plasmid-borne inserts (*Cbei_3974* and *Cbei_3904*) in *Cb* and furfural hampered phenotypic characterization of the resulting strains in furfural-challenged cultures (unpublished data). To circumvent this bottleneck, we explored genomic integration of both genes in *Cb* to eliminate the need for antibiotic supplementation, thereby allowing characterization of the resulting recombinant strains in furanic aldehyde- and phenolic compound-challenged cultures.

*Cbei_3974* and *Cbei_3904* were integrated into *Cb* genome and expressed under the control of a constitutive promoter (thiolase). Both genes were chromosomally integrated into *Cb* genome via double-cross homologous recombination to generate *Cb*_3974 (AKR) and *Cb*_3904 (SDR). Both strains were tested for the capacity to detoxify furfural and select lignin-derived microbial inhibitory compounds during ABE fermentation. Development of *Cb_*3974 *and Cb_*3904 represents a significant step towards fermentation of LB-derived sugars to biobutanol.

## Results

### Stable and functional integration of *Cbei_3974* (AKR) and *Cbei_3904* (SDR) into the genome of *C. beijerinc*kii NCIMB 8052

The primary goal of this study was to integrate and functionally express *Cbei_3974* (AKR) and *Cbei_3904* (SDR), both of which have been shown to play a role in furfural detoxification by *Cb* in our previous studies^1,16^, into the genome of *Cb* for improved detoxification of furfural and other LDMICs generated during pretreatment and hydrolysis of lignocellulosic biomass. To achieve this goal, we used the *Clostridium* integrative plasmid, pMTL-JH16, which targets *CA_C2872* (membrane protein) and *atpB* (F0/F1 ATP synthase subunit A) for replacement by homologous recombination^17^. Both *Cbei_3974* and *Cbei_3904* were placed under the control of a constitutive thiolase promoter from *Cb* to ensure expression of both genes from the inception of cell growth, which is critical for early and efficient detoxification of LDMICs in the culture broth. Upon successful integration of *Cbei_3974* (AKR) and *Cbei_3904* (SDR) in the *Cb* genome, both strains were characterized extensively relative to wild type *Cb* to test for stable expression of the integrated genes, cell growth, ABE production and detoxification of LDMICs. The growth profiles of *Cb_*3974 and *_*3904 were compared to the wild type. Interestingly, both strains (*Cb_*3974 and *_*3904) showed 1.2- and 1.3-fold increases in cell optical density, respectively, when compared to the wild type (Fig. S1).

Further, we conducted extensive stability test spanning 50 generations, which demonstrated the stability of both integrated genes in the genome of *Cb*. As shown in Figs S2–S4, butanol and ABE concentrations and cell optical densities did not decrease for either strain (*Cb_*3974 and *Cb_*3904) between generation zero (G_0_) and generation 50 (G_50_), following several sub-culturing. This confirms that, (a) the protein products of additional copies of both genes do not exert a deleterious effect on the growth of *Cb*, and (b) both enzymes do not disrupt cellular metabolism, particularly, the ABE fermentation pathway. Fifty generations were chosen to exceed the number of generations typically achieved in industrial-scale fermentations. Similarly, ethanol and acetone production were not affected in both strains after 50 generations (Figs S5b and S6b).

**Figure 1:**
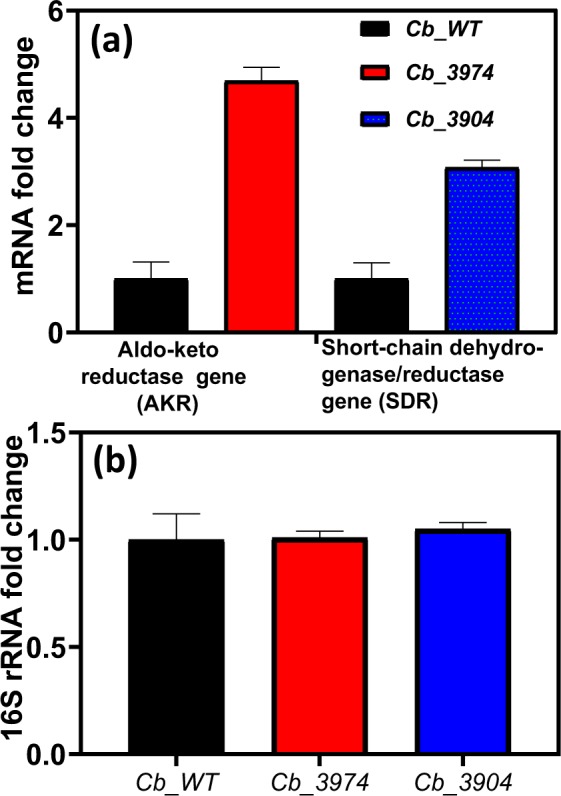
Fold changes in mRNA levels in *Cb*_3904 and _3974 strains relative to the wild type. (**a**) mRNA fold changes of *Cbei_3974* (AKR) and *Cbei_3904* (SDR) in *Cb*_3904 and _3974 relative to *Cb*_wild type. (**b**) mRNA fold changes of 16S rRNA gene in *Cb*_wild type, *Cb*_3904 and _3974.

**Figure 2:**
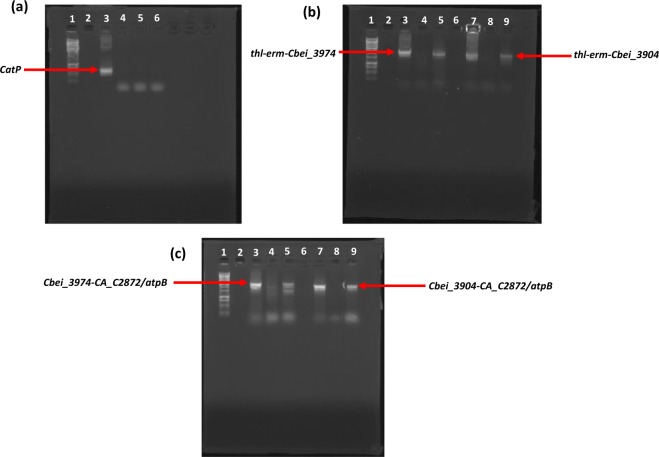
PCR amplification capturing the flanking regions of *Cbei_3974* and *Cbei_3904* following genomic integration, using gDNA from plasmid-cured *Cb*_3974 and *Cb*_3904 as templates. (**a**) Gel image showing *CatP* amplicon following PCR with *CatP*-gene specific primers. Lane 1: 1 kb DNA ladder, lanes 3, 4, 5 and 6: pMTL-JH16, gDNA from *Cb*_wildtype, *Cb*_3974 and *Cb*_3904, respectively. (**b**) Amplicons resulting from PCR amplification (step 1) of the flanking regions of chromosomally integrated *Cbei_3974* and *Cbei_3904* (*thl*-*erm*-*Cbei_*3974/*Cbei_3904*. DNA ladder (lane 1), pMTL-JH16_3974 (lane 3), *Cb*_wildtype (lane 4), *Cb*_3974 (lane 5), pMTL-JH16_3904 (lane 7), *Cb*_wild type (lane 8), *Cb*_3904 (lane 9). (**c**) Amplicons resulting from PCR amplification (step 2) of the flanking regions of chromosomally integrated *Cbei_3974* and *Cbei_3904* (*Cbei_*3974/*Cbei_*3904-*CA_C*2872/*atpB*). DNA ladder (lane 1), pMTL-JH16_3974 (lane 3), *Cb*_wild type (lane 4), *Cb*_3974 (lane 5), pMTL-JH16_3904 (lane 7), *Cb*_wild type (lane 8), *Cb*_3904 (lane 9). The gel images were cropped for clarity and the full-length gels are presented in Fig. S19.

To ascertain that *Cbei_3974* (AK*R*) and *Cbei_3904* (SDR) were expressed in *Cb* after integration, we conducted a quantitative real-time polymerase chain reaction (qRT-PCR) using specific primers for *Cbei_3974* and *Cbei_3904* (Table 1). Indeed, the mRNA levels for *Cbei_3974* (AKR) and *Cbei_3904* (SDR) increased 4.7- and 3-fold, respectively in *Cb*_3974 and _3904 relative to the wild type (Fig. 1). To further confirm stable integration of both genes, PCR was conducted using primers specific for the erythromycin resistance gene, which was part of the integration construct as a selectable marker. The erythromycin resistance gene was successfully amplified from generations zero to 50 (G_0_ to G_50_; Fig. S7a,b). Similarly, both strains of *Cb* [*Cb_*3974 (AKR) and *Cb_*3904 (SDR)] were plated out on erythromycin-un-supplemented and erythromycin-supplemented plates from G_0_ to G_50_ (Figs S8 and S9). Both strains exhibited dense growth on both erythromycin-supplemented and erythromycin-un-supplemented plates over 50 generations; expectedly, the wild type strain with no erythromycin resistance gene in the genome did not grow on plates containing erythromycin (Figs S8 and S9). Additionally, PCR was conducted using primers specific for the flanking regions of both integration constructs (*thl*-*erm*-*Cbei_3974*/*3904*-*CA_C2872*/*atpB*; Fig. 2b,c), using gDNA from *Cb*_3974 and *Cb*_3904 as templates. The wild type was used as negative control. To fully capture both constructs, PCR was conducted in two steps. In the first step, *thl*-*erm*-*Cbei_3974* or *thl*-*erm*-*Cbei_3974* was amplified (amplicon size: ~2400 kb and ~2300 kb for *C*b_3974 and *Cb*_3904, respectively; Fig. 2b). In the second step, *Cbei_3974*-*CA_C2872*/*atpB* or *Cbei_3904*-*CA_C2872*/*atpB* was captured (amplicon size: ~2400 kb and ~2300 kb for *C*b_3974 and *Cb*_3904, respectively; Fig. 2c). In each case, the integration construct was successfully confirmed by sequencing. Further, plasmid curing using mytomycin-C confirmed the absence of plasmids in both strains—*C*b_3974 and *Cb*_3904 (Fig. 2a). Additionally, the absence of *CatP* in both recombinant strains—*Cb*_3974 and *Cb*_3904—and its presence in the plasmid, pMTL-JH16, following PCR using gDNA from both strains and pMTL-JH16 as templates further confirmed genomic integration of *Cbei_3974* and *Cbei_3904* in the respective recombinant strains of *Cb*.

**Table 1:** List of PCR primers and PCR strategies. Underlined nucleotides (sequence) indicate ribosome binding sites that facilitate cloning of the *Cbei_3904* and *Cbei_3974.*

Primers	Target gene	Sequence (5′ → 3′)
***Cloning primers***		
Cb_3904 Forward	*Cbei_3904*	CTAAAT*GGTACC*AGGAGGAGTAAGGGCATGAGAAACTTAGAAGGTAAAGTTGCAATAATAACAGG
Cb_3904 Reverse	*Cbei_3904*	AAGAGTTACCATTTATTAAACATATCCACCATTAATTCGAAGAGTTTGACCTGTTATCC
Cb_3904 Reverse1	*Cbei_3904*	CAGGGCCTCGAGACTATGAAACAATATTAAAAAAATAAGAGTTACCATTTATTAAACATATCCACC
Cb_3974 Forward	*Cbei_3974*	CTAACT*GGTACC*AGGAGGGGCGGCGGCATGAAATATCAAGCATCAAAAAATAGATATAATGAGATG
Cb_3974 Reverse	*Cbei_3974*	CAATATTAAAAAAATAAGAGTTACCATTTACTAGTTAGCACTTATTTCATCAATCATCATCATTAACTC
Cb_3974 Reverse1	*Cbei_3974*	CAGGGC*CTCGAG*ACTATGAAACAATATTAAAAAAATAAGAGTTACCATTTACTAGTTAGC
Thl_pMTL-JH16 Forward	*thl*-*erm*-*Cbei*_3974 (or *Cbei*_3904)	TATGCAACAAAAGCAGCTATTGAAAAAGC
atpB Reverse	*Cbei*_3974 (or *Cbei*_3904)-*CA_C*2872/*atpB*	GCTGTAACACTAATATCCTTTGTTGCTGG
CatP Forward	*CatP*	ATGGTATTTGAAAAAATTGATAAAAATAGTTGG
CatP Reverse	*CatP*	TTAACTATTTATCAATTCCTGCAATTCG
***qRT-PCR primers***		
Cb_SDR F	*Cbei_3904*	GCTGTAGCTCCTGGTCCAAT
Cb_SDR R	*Cbei_3904*	CTGGTTCACCAATACGTCCA
Cb_AKR F	*Cbei_3974*	CCAGACGATAGCCGGATAAA
Cb_AKR R	*Cbei_3974*	TGAGCAAGAGTTTGCCCTCT
16SrRNA F	*16S rRNA*	GAAGAATACCAGTGGCGAAGGC
16SrRNA R	*16S rRNA*	ATTCATCGTTTACGGCGTGGAC

### Fermentation profiles of furfural-challenged cultures of *Cb*_3974, *Cb*_3904 and *Cb*_wild type

#### Growth

**Figure 3:**
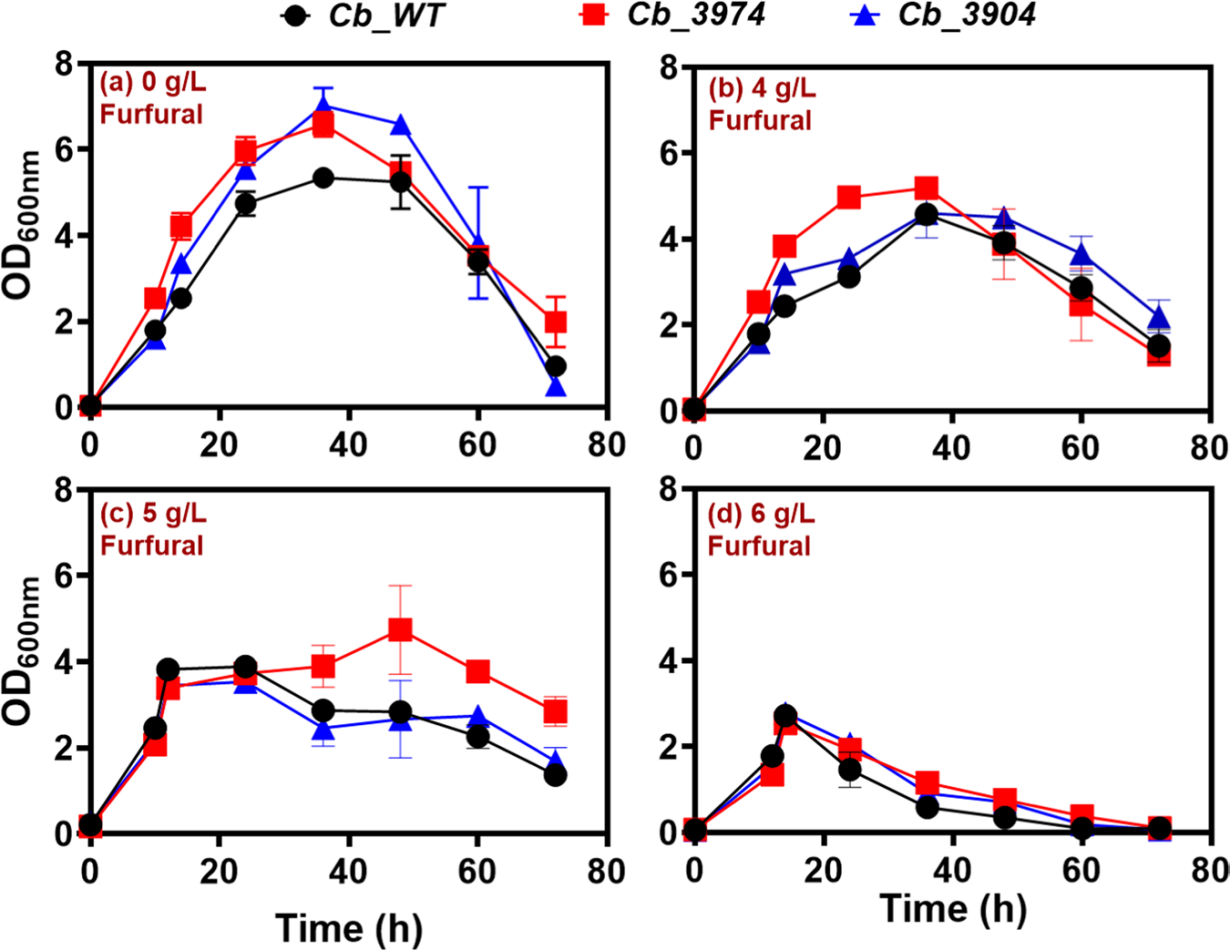
The growth profiles of *Cb*_3974, _3904 and _wild type in furfural (0–6 g/L)-challenged cultures.

The cultures of *Cb*_3974, *Cb*_3904 and *Cb*_wild type were challenged with furfural (0, 4, 5, and 6 g/L) in fermentation medium and optical density and the concentrations of butanol, acetone, ethanol, and ABE were measured. In addition, the rate of furfural detoxification was monitored by measuring the concentrations of furfural and furfuryl alcohol—the less toxic product of furfural reduction—in the fermentation broth. In each case, furfural was added to the cultures after an optical density of 2.0 had been attained (10–12 hours fermentation). Overall, *Cb*_3974 showed greater capacity to detoxify furfural when compared to *Cb*_3904 and *Cb*_wild type. With 0 g/L furfural, the optical densities of *Cb*_3974 and *Cb*_3904 were 1.2- and 1.3-fold greater than that of *Cb*_wild type (Fig. 3a). With 4 g/L furfural, *Cb*_3974 showed a higher rate of cell biomass accumulation, reaching a maximum optical density of 5.2, which is 1.1-fold higher than those of *Cb*_3904 and *Cb*_wild type—both of which exhibited a similar growth profile (Fig. 3b). Notably, when compared to 0 g/L furfural, the optical densities of both *Cb*_3974 and *Cb*_3904 reduced considerably upon challenge with 4 g/L furfural. However, the reduction in growth was more pronounced with *Cb*_3904, in which 1.5-fold reduction in optical density was observed, relative to 1.3-fold reduction in optical density for *Cb*_3974. The robust ability of *Cb*_3974 to tolerate furfural relative to *Cb*_3904 and *Cb*_wild type is more clearly highlighted by the optical densities of all three strains of *Cb* challenged with 5 g/L furfural (Fig. 3c). With 5 g/L furfural, *Cb*_3974 reached a maximum optical density of 4.7, which is 1.2- and 1.3-fold greater than the maximum optical densities reached by *Cb*_3904 and *Cb*_wild type, respectively. Again, as with 4 g/L furfural, both *Cb*_3904 and *Cb*_wild type exhibited a similar growth profile in cultures supplemented with 5 g/L furfural (Fig. 3c). A concentration of 6 g/L furfural proved deleterious to all three strains of *Cb*, which exhibited a similar growth profile (Fig. 3d). In fact, with 6 g/L furfural, after achieving maximum optical densities of 2.72, the optical density of *Cb*_wild decreased 1.3- and 1.4-fold, relative to the optical densities of *Cb*_3974 and *Cb*_3904, respectively.

#### Furfural detoxification

**Figure 4:**
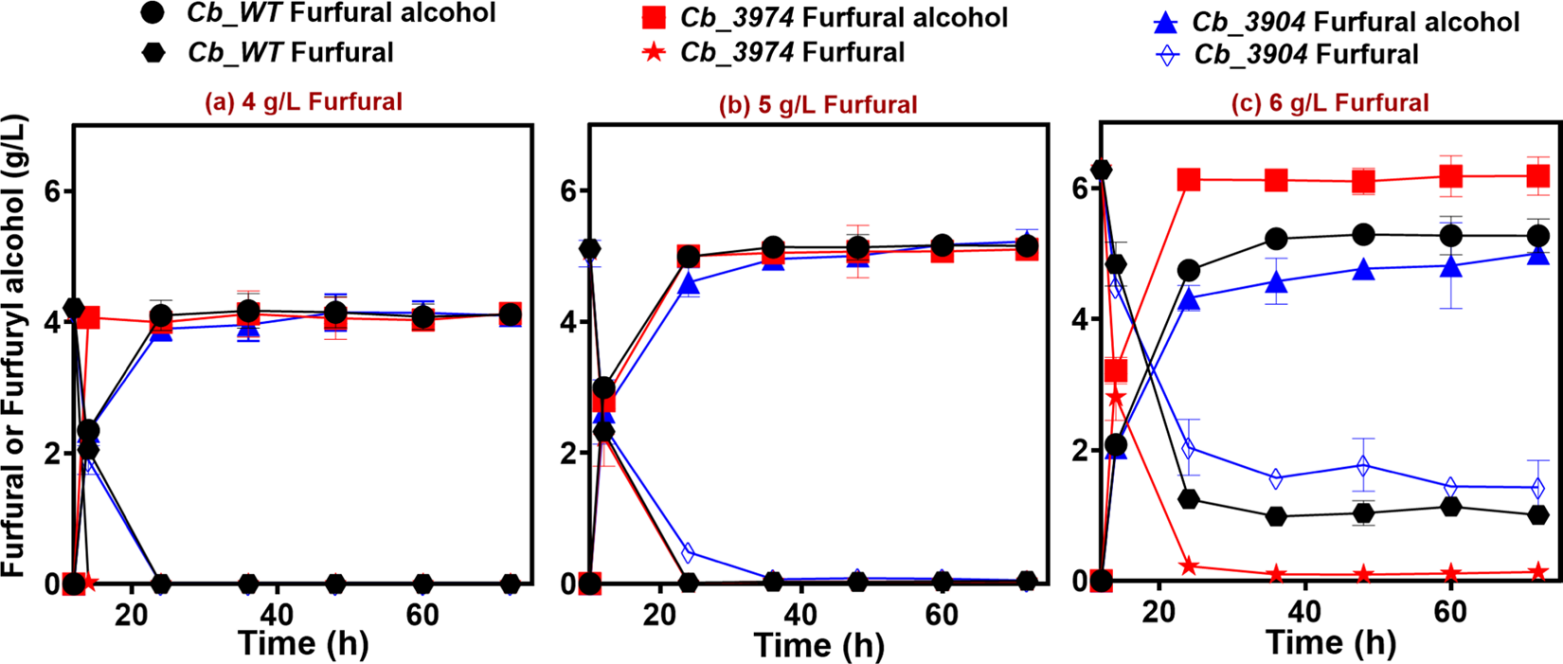
Furfural detoxification profiles of *Cb*_3974, _3904, and _wild type in furfural (0–6 g/L)-challenged cultures.

The furfural detoxification profiles of *Cb*_3974, *Cb*_3904 and *Cb*_wild type underscore the superior capability of *Cb*_3974 [expressing an additional copy of the aldo-keto reductase (AKR) gene, *Cbei_3974*] to reduce furfural to the less toxic furfuryl alcohol, when compared to *Cb*_3904 and *Cb*_wild type. With 4 g/L furfural, *Cb*_3974 exhibited a furfural detoxification rate of 2.0 g/L/h, whereas the detoxification rates for *Cb*_3904 and *Cb*_wild type were ~1.1 g/L/h and 1.0 g/l/h, respectively (Fig. 4a). This represents a ~2-fold higher rate of furfural detoxification by *Cb*_3974 relative to *Cb*_3904 and _wild type. With 5 g/L furfural, all three strains showed similar rates of furfural detoxification (0.23 g/L/h for *Cb*_3974; ~0.22 g/L/h for *Cb*_3904 and _wild type; Fig. 4b). When challenged with 6 g/L furfural, this concentration exerted a far greater toxic effect on all three strains of *Cb*. Nonetheless, *Cb*_3974 showed a more robust capacity to detoxify furfural at this concentration. With 6 g/L furfural, the rates of furfural detoxification for *Cb*_3974, _3904 and _wild type were ~0.1 g/L/h, ~0.07 g/L/h, and ~0.08 g/L/h, respectively (Fig. 4b). This represents 1.4- and 1.25-fold faster rates of detoxification by *Cb*_3974 when compared to *Cb*_3904 and _wild type.

#### Glucose utilization

As depicted in Table 2, all three strains (*Cb*_3974, _3904, and _wild type) consumed relatively the same amount of glucose (~56 g/L) in cultures un-supplemented with furfural (0 g/L). The rate of glucose utilization in the 0 g/L furfural-supplemented cultures was 0.8 g/L/h for all the three strains studied. With 4 g/L furfural challenge however, glucose utilization reduced considerably for all the strains studied. Notably, reduction in glucose utilization was more pronounced in cultures of *Cb*_3904. Following 4 g/L furfural challenge, *Cb*_3974, _3904 and _wild type consumed ~54 g/L, ~41 g/L, and ~52 g/L glucose, respectively, which translates to ~1.4-, and ~1.1-fold decreases in glucose utilization by *Cb*_3904 and _wild type, respectively (Table 2), when compared to the cultures challenged with 0 g/L furfural. The rates of glucose utilization in the 4 g/L furfural-supplemented cultures were 0.75 g/L/h, 0.57 g/L/h, and 0.72 g/L/h in cultures of *Cb*_3974, _3904, and _wild type, respectively. Increasing furfural concentration to 5 g/L led to marked reduction in glucose utilization for all the three strains of *Cb*. The total glucose consumed by *Cb*_3974, _3904, and _wild type when challenged with 5 g/L furfural were ∼27 g/L, 13.2 g/L, and 17.3 g/L, respectively, which amount to 2.1-, 4.2- and 3.2-fold decreases in glucose consumption relative to furfural un-supplemented cultures (Table 2). Consequently, the rates of glucose utilization by *Cb*_3974, _3904, and _wild type reduced significantly (~0.37 g/L/h, 0.20 g/L/h, and ~0.24 g/L/h, respectively). When 6 g/L furfural was pulse-fed into the culture medium, *Cb*_3974, _3904, and _wild type consumed 25.7 g/L, 14.9 g/L and 17.0 g/L of glucose, respectively, which represent ~2.1-, 3.7-, and 3.3-fold decreases in glucose consumption, respectively (Table 2). Accordingly, the rate of glucose utilization by *Cb*_3974 when challenged with 6 g/L furfural further reduced slightly to ~0.36 g/L/h, when compared to cultures challenged with 5 g/L furfural. Conversely, the rate of glucose utilization by *Cb*_3904 (0.20 g/L/h) and *Cb*_wild type (0.24 g/L/h) did not decrease any further following 6 g/L furfural challenge, when compared to 5 g/L furfural-challenged cultures in which the rates of glucose utilization were 0.20 g/L/h and 0.24 g/L, respectively. Furfural toxicity appeared to have plateaued at 5 g/L for both strains (*Cb*_3904 and *Cb*_wild type), such that when furfural concentration was increased to 6 g/L, no further decrease in glucose utilization occurred.

**Table 2:** Summary of the fermentation profiles of *Cb*_3974, *Cb*_3904 and *Cb*_wild type in Furfural (0–6 g/L)-challenged cultures.

Treatments		_wild type	_3974	_3904
0 g/L Furfural	Glucose consumed (g/L)	56.0 ± 0.8	57.6 ± 0.6	55.7 ± 9.0
	Residual glucose (g/L)	8.1 ± 0.2	1.7 ± 0.4	3.2 ± 0.4
	Max. Butanol (g/L)	11.2 ± 1.2	12.5 ± 0.1	10.9 ± 1.3
	Max. ABE (g/L)	16.0 ± 2.0	17.9 ± 0.2	16.7 ± 2.4
	ABE yield (g/g)	0.3 ± 0.0	0.3 ± 0.0	0.3 ± 0.0
	ABE productivity (g/L/h)	0.3 ± 0.0	0.3 ± 0.0	0.3 ± 0.0
4 g/L Furfural	Glucose consumed (g/L)	52.1 ± 0.8	53.7 ± 0.3	41.47 ± 6.9
	Residual glucose (g/L)	11.9 ± 1.4	5.6 ± 0.6	17.5 ± 1.1
	Max. Butanol (g/L)	9.2 ± 0.8	12.2 ± 0.1	9.2 ± 0.3
	Max. ABE (g/L)	15.3 ± 1.6	18.9 ± 0.0	15.6 ± 0.4
	ABE yield (g/g)	0.3 ± 0.0	0.4 ± 0.0	0.4 ± 0.0
	ABE productivity (g/L/h)	0.3 ± 0.0	0.4 ± 0.0	0.3 ± 0.0
5 g/L Furfural	Glucose consumed (g/L)	17.3 ± 4.2	26.8 ± 1.3	13.2 ± 1.3
	Residual glucose (g/L)	46.9 ± 2.8	38.3 ± 1.5	52.3 ± 0.7
	Max. Butanol (g/L)	1.2 ± 0.1	5.4 ± 1.0	1.3 ± 0.3
	Max. ABE (g/L)	2.7 ± 0.1	9.2 ± 1.6	3.2 ± 0.4
	ABE yield (g/g)	0.2 ± 0.0	0.4 ± 0.0	0.3 ± 0.1
	ABE productivity (g/L/h)	0.1 ± 0.0	0.2 ± 0.0	0.1 ± 0.0
6 g/L Furfural	Glucose consumed (g/L)	17.0 ± 2.2	25.7 ± 2.3	14.9 ± 0.6
	Residual glucose (g/L)	48.0 ± 0.8	35.6 ± 3.5	46.8 ± 2.4
	Max. Butanol (g/L)	1.0 ± 0.1	4.5 ± 0.5	1.0 ± 0.1
	Max. ABE (g/L)	3.8 ± 0.5	9.6 ± 1.9	4.6 ± 0.0
	ABE yield (g/g)	0.2 ± 0.1	0.4 ± 0.0	0.3 ± 0.0
	ABE productivity (g/L/h)	0.1 ± 0.0	0.2 ± 0.0	0.1 ± 0.0

#### ABE production

**Figure 5:**
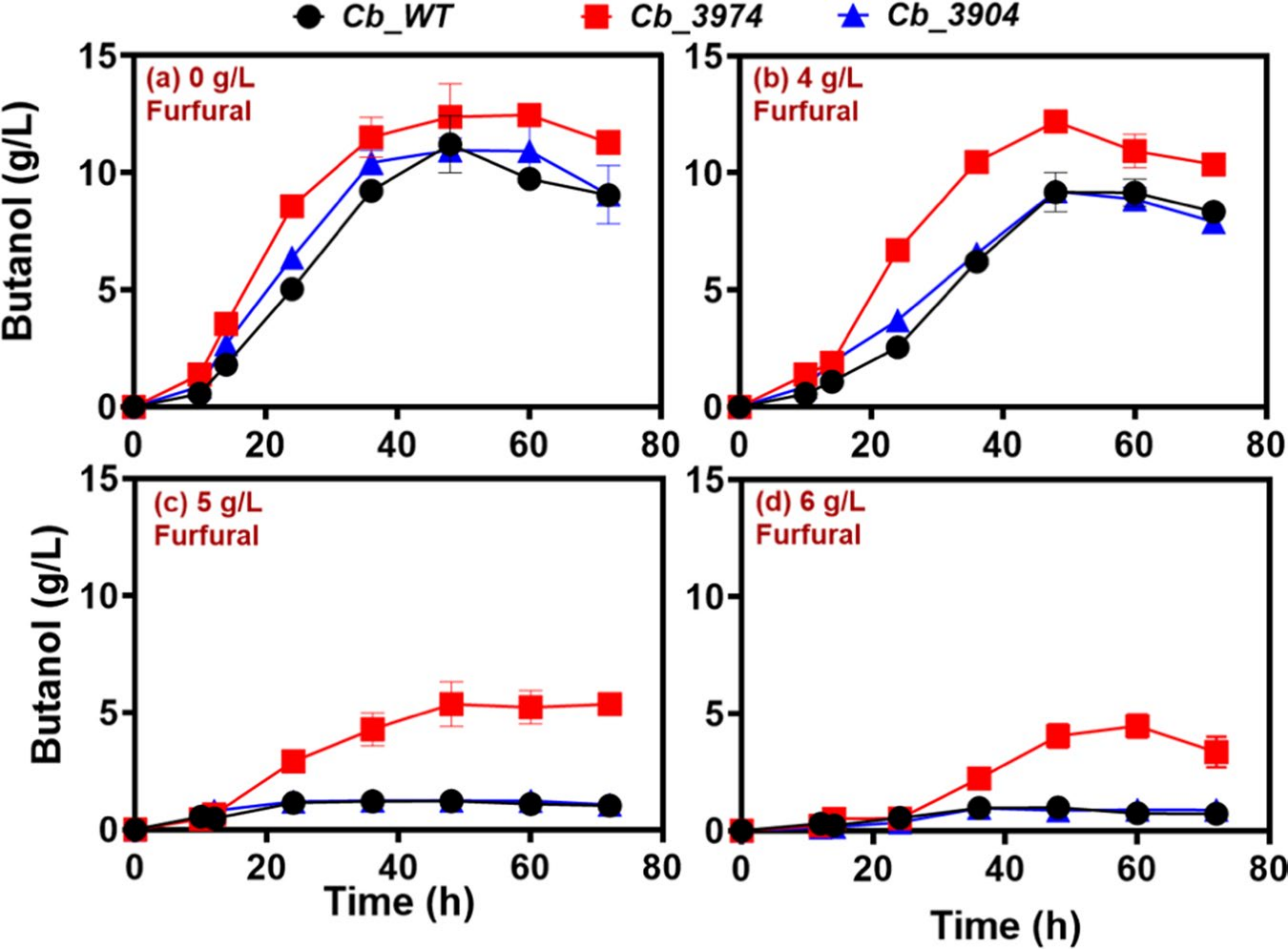
Butanol production by *Cb*_3974, _3904, and _wild type in P2 fermentation medium supplemented with 0–6 g/L furfural.

**Figure 6:**
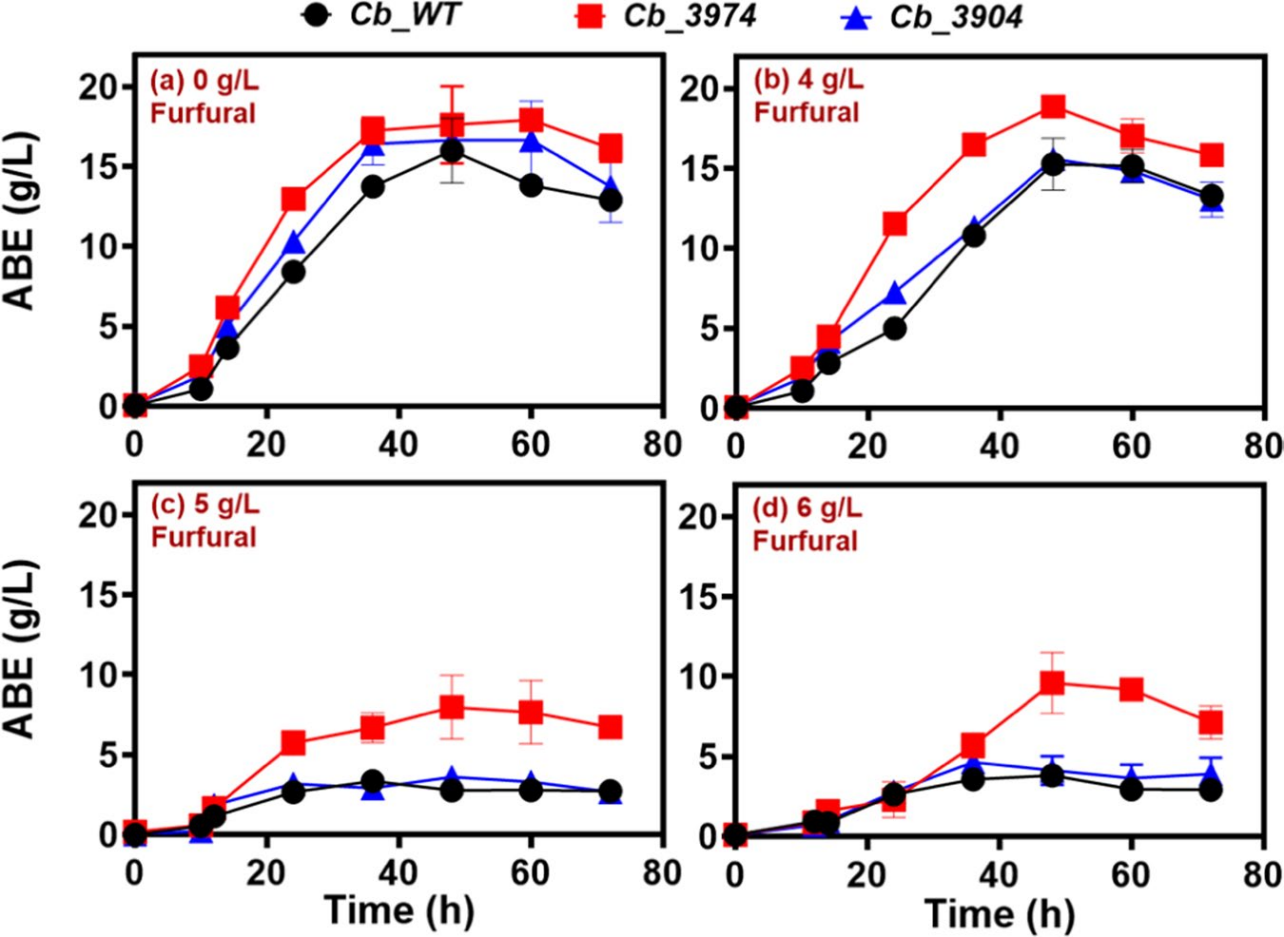
ABE profiles of *Cb*_3974, _3904, and _wild type in P2 fermentation medium supplemented with 0–6 g/L furfural.

At all concentrations of furfural tested, *Cb*_3974 produced considerably more butanol than *Cb*_3904 and *Cb*_wild type (Fig. 5). In fact, with 0 g/L furfural, *Cb*_3974 produced 12.5 g/L butanol, which is 1.2- and 1.1-fold greater than the maximum butanol concentrations produced by *Cb*_3904 (10.9 g/L butanol) and *Cb*_wild type (11.2 g/L butanol; Fig. 5a), respectively. With 4 g/L furfural, *Cb*_3974 produced 12.2 g/L butanol, whereas each of *Cb*_3904 and *Cb*_wild type produced 9.2 g/L (Fig. 5b), which represents 1.3-fold higher butanol concentration in cultures of *Cb*_3974 relative to *Cb*_3904 and _wild type. Similarly, when challenged with 5 g/L furfural, *Cb*_3974 produced 5.4 g/L butanol, which is 4.5- and 4.2-fold greater than the concentrations produced by *Cb*_3904 (1.2 g/L butanol) and *Cb*_wild type (1.3 g/L butanol; Fig. 5c), respectively. With 6 g/L furfural, *Cb*_3974 produced 4.5 g/L butanol, whereas *Cb*_3904 and *Cb*_wild type each produced 1.0 g/L butanol (Fig. 5d). The butanol concentration in cultures of *Cb*_3974 was ~4.5-fold greater than the concentrations produced by *Cb*_3904 and _wild type, respectively, when fermentation was supplemented with 6 g/L furfural. The ABE profiles of *Cb*_3974, _3904 and _wild type are congruent with the butanol profiles at all the concentrations of furfural tested—*i.e*. *Cb*_3974 showed greater capacity to accumulate ABE following furfural challenge (Fig. 6). When fermentation cultures were supplemented with 0 g/L furfural, *Cb*_3974 produced marginally higher ABE concentration (18.0 g/L), when compared to *Cb*_3904 (17.0 g/L; 1.05-fold lower than *Cb*_3974) and *Cb*_wild type [(16.0 g/L; 1.13-fold lower than *Cb*_3974); Fig. 6a]. In cultures supplemented with 4 g/L furfural, *Cb*_3974 produced 19.0 g/L ABE, which is 1.2-fold greater than the concentrations produced by *Cb*_3904 (15.6 g/L) and _wild type (15.3 g/L). Similarly, when fermentation cultures were challenged with 5 g/L furfural, the ABE concentration in cultures of *Cb*_3974 (9.2 g/L) was ~3.0- and 3.4-fold greater than the concentrations produced by *Cb*_3904 (3.2 g/L) and _wild type (2.7 g/L). With 6 g/L furfural, *Cb*_3974 produced 9.6 g/L ABE; which is 2.1- and ~2.5-fold greater than the concentrations produced by *Cb*_3904 (4.6 g/L) and *Cb*_wild type (3.8 g/L). Likewise, *Cb*_3974 accumulated greater acetone concentration (4.5 g/L) than *Cb*_3904 (4.4 g/L) and _wild type (3.9 g/L), albeit only in cultures challenged with 4 g/L furfural (Fig. S10).

### Effects of lignin-derived inhibitory compounds on ABE fermentation by *Cb*_3974, *Cb*_3904 and *Cb*_wild type

#### Growth

Although lignin-derived microbial inhibitory compounds occur at lower concentrations than furanic aldehydes (furfural and HMF) in lignocellulosic biomass hydrolysates, they exert significant toxic effects on fermenting microorganisms. This stems in part from the fact that there are far more lignin-derived inhibitory compounds in biomass hydrolysates than furanic aldehydes, which collectively represent a formidable threat to microorganisms during fermentation. In this study, we tested the effects of 4-hydroxybenzaldehyde (4-HBD), syringaldehyde, syringic acid and vanillic acid (0.5, 0.75, and 1.0 g/L) on *Cb*_3974, *Cb*_3904 and *Cb*_wild type. At the concentrations tested (0.5, 0.75, and 1.0 g/L), supplementation of the culture medium with 4-HBD, syringaldehyde, syringic acid, and vanillic acid did not lead to significant changes in the optical densities of *Cb*_3974 and *Cb*_3904 relative to *Cb*_wild type (data not shown).

#### ABE production

**Figure 7:**
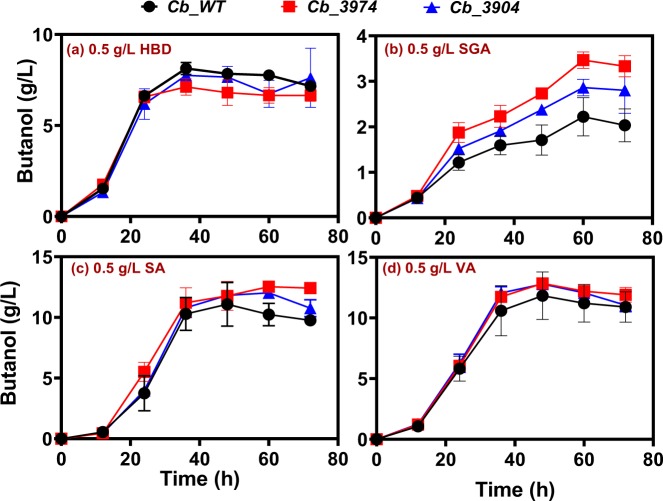
Butanol concentrations produced *Cb*_3974, _3904, and _wild type during ABE fermentation supplemented with 0.5 g/L 4-hydroxybenzaldehyde (4-HBD), syringaldehyde (SGA), syringic acid (SA), and vanillic acid (VA).

*Cb*_3974, _3904 and _wild type exhibited similar butanol profiles when challenged with 0.5 g/L of the lignin-derived inhibitors tested, with the exception of syringaldehyde. With 0.5 g/L syringaldehyde treatment, *Cb*_3974 produced ~3.5 g/L butanol, which represents 1.2- and 1.6-fold increases in butanol concentration relative to *Cb*_3904 (2.93 g/L) and *Cb*_wild type (2.22 g/L), respectively (Fig. 7). Notably, among the four lignin-derived inhibitors studied (4-HBD, syringaldehyde, syringic acid, and vanillic acid), syringaldehyde had the most marked impact on butanol production. For instance, with 0.5 g/L 4-HBD, syringic acid, and vanillic acid, each of *Cb*_3974, _3904 and _wild type produced at least 7.12, 11.10, and 11.80 g/L butanol. On the other hand, the maximum butanol produced with 0.5 g/L syringaldehyde (3.5 g/L produced by *Cb*_3974) was at least 2-fold lower than the butanol concentration produced in cultures challenged with 0.5 g/L 4-HBD, syringic acid or vanillic acid (Fig. 7). In fact, the maximum butanol concentration produced by *Cb*_wild type with 0.5 g/L syringaldehyde was ~3.7-fold lower than the maximum butanol concentration (12.85 g/L produced by *Cb*_3974 challenged with vanillic acid) produced by any of the three strains challenged with 0.5 g/L of 4-HBD, syringic acid or vanillic acid.

The ABE profiles of *Cb*_3974, _3904 and _wild type challenged with 0.5 g/L 4-HBD, syringic acid, and vanillic acid were comparable, whereas *Cb*_3974 challenged with syringaldehyde produced 1.2- and 1.3-fold greater ABE than *Cb*_3904 and *Cb*_wild type exposed to syringaldehyde stress (0.5 g/L; Fig. S11). As with butanol, syringaldehyde exerted a considerably higher effect on ABE production, when compared to 4-HBD, syringic acid, and vanillic acid. The maximum ABE produced with 0.5 g/L syringaldehyde (7.77 g/L by *Cb*_3974) was at least, 1.6-fold lower than the lowest ABE concentration (12.05 g/L by *Cb*_3974 challenged with 4-HBD) produced with 4-HBD, syringic acid or vanillic acid (Fig. S11).

**Figure 8:**
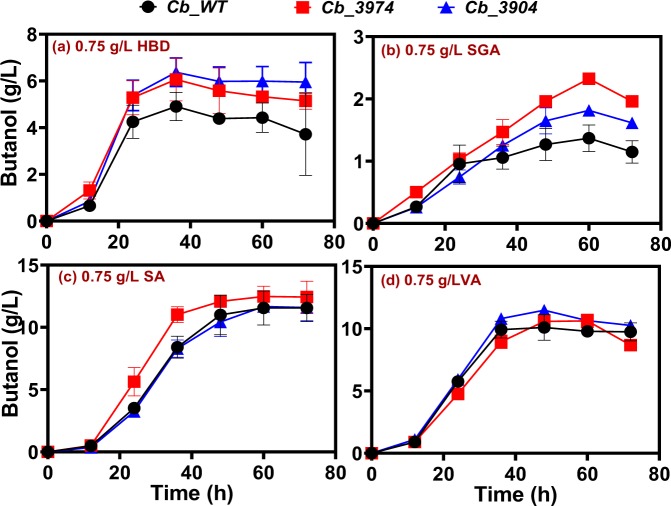
Butanol concentrations produced by *Cb*_3974, _3904, and _wild type during ABE fermentation in cultures supplemented with 0.75 g/L 4-hydroxybenzaldehyde (4-HBD), syringaldehyde (SGA), syringic acid (SA), and vanillic acid (VA).

When the concentrations of 4-HBD, syringaldehyde, syringic acid, and vanillic acid were increased to 0.75 g/L, *Cb*_3974 demonstrated greater capacity to tolerate 4-HBD, syringaldehyde, and syringic acid but not vanillic acid (Fig. 8). For instance, with 0.75 g/L 4-HBD, *Cb*_3974 and *Cb*_3904 showed a similar pattern of butanol accumulation. At this concentration of 4-HBD (0.75 g/L), *Cb*_3974 and *Cb*_3904 produced 6.06 and 6.37 g/L butanol, respectively, which were 1.2- and 1.3-fold greater than the butanol concentration produced by *Cb*_wild type (Fig. 8a). Further, cultures of *Cb*_3974 supplemented with 0.75 g/L syringaldehyde produced 1.3- and 1.7-fold more butanol than *Cb*_3904 and *Cb*_wild type, respectively (Fig. 8b), whereas 0.75 g/L syringic acid supplementation resulted in 1.1-fold greater butanol production by *Cb*_3974 relative to *Cb*-wild type. Both *Cb*_3974 and *Cb*_3904 exhibited similar butanol profiles with 0.75 g/L syringic acid supplementation (Fig. 8c). Supplementation of culture medium with 0.75 g/L vanillic acid did not result in any significant differences in butanol concentration among the three strains studied (Fig. 8d). Greater butanol production by *Cb*_3974 in fermentations supplemented with 0.75 g/L 4-HBD, syringaldehyde, and syringic acid led to higher concentrations of ABE, particularly when compared to *Cb*_wild type challenged with 0.75 g/L 4-HB, syringaldehyde, and syringic acid (Fig. S12). For example, ABE concentrations in cultures of *Cb*_3974 challenged with 0.75 g/L4-HBD, syringaldehyde, and syringic acid were 1.2-fold higher than those of *Cb*_wild type challenged with each of 4-HBD, syringaldehyde, and syringic acid (0.75 g/L; Fig. S12a–c). In part, this was as a result of superior acetone production by *Cb*_3974 relative to the wild type (data not shown). Interestingly, although 0.75 g/L vanillic acid supplementation did not elicit significantly greater butanol production by *Cb*_3974 when compared to the wild type, ABE production was 1.1-fold higher in cultures of *Cb*_3974 when compared to *Cb*_wild type (Fig. S12d). Similarly, this stemmed from greater acetone production (1.2-fold higher) by *Cb*_3974 relative to the *Cb*_wild type. Conversely, increased butanol production by *Cb*_3974 relative to *Cb*_3904 (when both strains were supplemented with 0.75 g/L syringaldehyde and syringic acid), did not translate into considerably greater ABE concentrations in the cultures of *Cb*_3974 when compared to *Cb*_3904 (Fig. S12). This was due to greater acetone and/or ethanol production by *Cb*_3904 when compared to the cultures of *Cb*_3974 (data not shown), which offset the higher butanol concentrations observed in cultures of *Cb*_3974.

**Figure 9:**
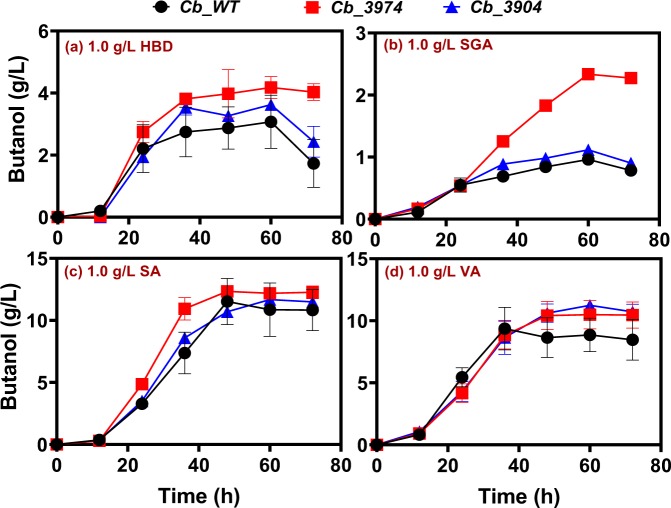
Butanol concentrations in cultures of *Cb*_3974, _3904, and _wild type during ABE fermentations supplemented with 1.0 g/L 4-hydroxybenzaldehyde (4-HBD), syringaldehyde (SGA), syringic acid (SA), and vanillic acid (VA).

Lastly, we tested the effects of 1.0 g/L 4-HBD, syringaldehyde, syringic acid, and vanillic acid on the ABE profiles of *Cb*_3974, _3904, and _wild type. Notably, when the concentration of 4-HBD increased to 1.0 g/L, the acetone concentrations of *Cb*_3974, _3904 and _wild type mirrored one another, ranging from 1.56–1.85 g/L (Fig. S13a). On the other hand, *Cb*_3974 produced 1.4- and 1.2-fold greater butanol concentrations than *Cb*_wild type and _3904, respectively (Fig. 9a), and 1.2- and 1.1-fold greater ABE concentrations than *Cb*_wild type and _3904, respectively (Fig. S14a). With 1.0 g/L syringaldehyde, acetone concentrations in the cultures of *Cb*_3974 were 3.8- and 2.1-fold greater than those of *Cb*_wild type and _3904, respectively (Fig. S13b). Similarly, butanol concentrations in the cultures of *Cb*_3974 supplemented with1.0 g/L syringaldehyde were 2.4- and 2.1-fold higher than those of *Cb*_wild type and _3904, respectively, supplemented with 1.0 g/L syringaldehyde (Fig. 9b), whereas the ABE concentrations of *Cb*_3974 were 1.4-fold greater than those of *Cb*_wild type and _3904 (Fig. S14b). With 1.0 g/L syringic acid and vanillic acid, *Cb*_3974 and _3904 showed similar butanol and ABE profiles (Figs 9c,d and S14c,d). Notably, *Cb*_3974 exhibited greater productivity (1.2- and 1.3-fold for butanol and 1.2- and 1.5-fold for ABE) than *Cb*_3904 in vanillic acid- and syringic acid (1.0 g/L)-supplemented cultures (Figs 9c,d and S14c,d). Further, butanol and ABE concentrations in the cultures of *Cb*_3974 supplemented with 1.0 g/L vanillic acid were 1.1- and 1.2-fold greater, respectively, when compared to the cultures of *Cb*_wild type subjected to the same treatments (Figs 9d and S14d). Likewise, butanol concentrations in the cultures of *Cb*_3974 supplemented with 1.0 g/L syringic acid were ~1.1- higher than those in cultures of *Cb*_wild type exposed to 1.0 g/L syringic acid (Fig. 9c). However, the ABE concentrations in both sets of cultures (*Cb*_3974 and *Cb*_wild type supplemented with 1.0 g/L syringic acid) were considerably similar (Fig. S14c) due to varying concentrations of acetone, and in some cases ethanol.

### The relative toxicities of lignin-derived inhibitors (4-HBD, syringaldehyde, syringic acid and vanillic acid) on *Cb*_3974, *Cb*_3904, and *Cb*_wild type

**Figure 10:**
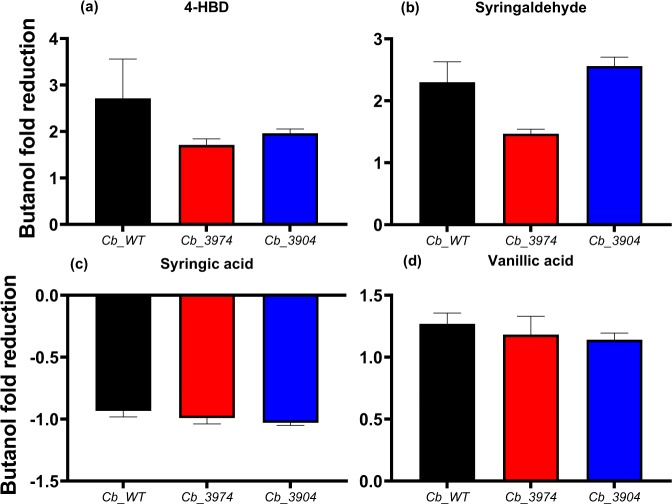
Fold reductions in butanol concentrations as a measure of relative toxicities of 4-HBD, syringaldehyde, syringic acid, and vanillic acid on *Cb*_3974, _3904, and _wild type.

LDMICs disrupt solventogenesis in solventogenic *Clostridium* species by means of multifarious mechanisms^1,18,19^. In addition, the toxicity of butanol in solventogenic *Clostridium* species increases with increasing concentration of LDMICs. As a result, when grown in lignocellulosic hydrolysates (which typically contain LDMICs), butanol production by solventogenic clostridia reduces drastically, relative to the other solvents (acetone and ethanol)^20^. The flux towards acetone and ethanol production likely reduces butanol production and combined toxicities of butanol and LDMICs in lignocellulosic hydrolysates and fermentation media containing model LDMICs. Therefore, a measure of fold reduction in butanol production over increasing inhibitor concentrations can serve as a measure of the degrees of toxicity exerted by different LDMICs on solventogenic *Clostridium* species. More importantly, the relative toxicities of these inhibitors on different strains reflect the tolerance capacities of the strains against the tested inhibitory compounds. Figure 10 depicts the fold reductions in butanol concentrations in cultures of *Cb*_3974, _3904, and _wild type challenged with increasing concentrations (0.5–1.0 g/L) of 4-HBD, syringaldehyde, syringic acid, and vanillic acid (lignin-derived microbial inhibitory compounds). Clearly, *Cb*_3974 is the most tolerant to the lignin-derived inhibitors tested, relative to *Cb*_3904 and _wild type. For example, butanol concentrations reduced 2.7-, 1.9-, and 1.7-fold, respectively in cultures of *Cb*_wild type, _3904, and _3974, when 4-HBD concentration in the fermentation medium was increased from 0.5 to 1.0 g/L (Fig. 10a). Similarly, increasing syringaldehyde concentration from 0.5 to 1.0 g/L resulted in 2.3-, 2.6-, and 1.5-fold reductions in butanol concentrations, respectively, in cultures of *Cb*_wild type, _3904, and _3974 (Fig. 10b). With syringic acid, no reductions in butanol concentration were observed for all three strains studied following increase in concentration from 0.5 to 1.0 g/L (Fig. 10c). Conversely, increasing syringic acid concentration resulted in increased butanol production. With vanillic acid, the fold reductions in butanol concentration were similar for all three strains: 1.3-, 1.1-, and 1.2-fold, respectively for *Cb*_wild type, _3904, and _3974, following increase in concentration from 0.5 to 1.0 g/L (Fig. 10d). Clearly, the aldehydes (4-HBD and syringaldehyde) exerted significantly greater negative effect on butanol production than the phenolic acids—vanillic and syringic acids.

## Discussion

Having previously cloned, purified and characterized the aldo-keto reductase (AKR) encoded by the gene, *Cbei_3974*, and the short chain dehydrogenase/reductase (SDR) encoded by *Cbei_3904* in *E. coli*^1^, in this study we investigated the feasibility of functionally integrating both genes (separately) into the genome of *Cb*, and evaluated tolerance of the resulting strains to LDMICs. To circumvent the combined toxicities of erythromycin and LDMICs on *Cb* during fermentation, both genes were homologously integrated into the *Cb* genome, thereby excluding the need for erythromycin supplementation for plasmid maintenance. Whereas both *Cb*_3974 and _3904 showed greater tolerance to most of the inhibitory compounds tested when compared to the wild type, by far *Cb*_3974 demonstrated superior inhibitor tolerance than *Cb*_3904. This, most plausibly stems from the contrasting enzymatic characteristics of both enzymes. In a previous study, we demonstrated that AKR exhibited 7.1-fold greater specific activity on furfural than the SDR^1^. Similarly, AKR was found to be significantly more active than SDR on 4-hydroxybenzaldehyde and 5-hydroxymethyl furfural (HMF). Although both AKR and SDR were strongly upregulated in furfural-challenged *Cb*^16^, the growth and ABE profiles of *Cb*_3974 and _3904 (particularly greater accumulation of butanol in cultures of *Cb*_3974 challenged with inhibitors relative to those of _3904 and _wild type) indicate that AKR likely plays significant primary role in *in situ* detoxification of LDMICs by *Cb*. Conversely, SDR likely plays a secondary role in LDMICs tolerance by *Cb*.

Interestingly, the protein product of *Cbei_3904* (SDR) has recently been re-annotated as a tetrahydroxynaphthalene/trihydroxynaphthalene reductase-like enzyme involved in fatty acid biosynthesis and co-factor (biotin) metabolism^21,22^. Both functions—fatty acid biosynthesis and biotin metabolism—are relevant in furfural tolerance; hence, one or both metabolic tasks may represent the role of SDR during furfural challenge. For instance, biotin is an important bacterial co-factor whose intracellular demand might increase significantly following up-regulation of otherwise not expressed multiple biotin-dependent proteins involved in furfural tolerance or detoxification. Notably, *Cbei_4439* which encodes a biotin and thiamin synthesis-associated protein was also strongly up-regulated (~5-fold) in furfural-challenged *Cb*^16^. Similarly, *Cbei_1224*, *Cbei_1225* and *Cbei_1227,* which encode proteins involved in riboflavin biosynthesis—another co-factor—were up-regulated 9.2-, 9.1-, and 6.8-fold, respectively, in furfural-challenged *Cb*^16^. Likewise, changes in fatty acid metabolism have been implicated in LDMICs tolerance. Specifically, Levulinic, acetic and cinnamic acids, furfural, and phenol have been shown to elicit modulation of cell lipid composition in *S. cerevisiae*, leading to increased tolerance to these inhibitors^23,24^. Additionally, *S. cerevisiae* typically modulates lipid composition to increase membrane integrity when exposed to membrane-damaging stressors^25^. Among other effects, LDMICs induce membrane damage during onslaught on fermenting microbial cells^16,19,26^. This leads to modulation of lipid composition by inhibitor-challenged cells, in an attempt to strengthen membrane integrity. These (increased biotin demand and membrane lipid modulation) may account for up-regulation of *Cbei_3904* (SDR) in furfural-challenged *Cb*^16^.

For each strain studied, among the lignin-derived inhibitory compounds (phenolic compounds) tested, the aldehydes—4-HBD and syringaldehyde—were significantly more toxic than the phenolic acids (syringic acid and vanillic acid; Fig. 10). In fact, syringic acid supplementation increased butanol production, whereas vanillic acid supplementation led to marginal decrease in growth (data not shown) and butanol production (Figs 6–9). Notably, syringaldehyde was considerably more toxic, exerting considerably greater inhibition on both growth and solvent biosynthesis than 4-HBD (Figs 6–8). This is in agreement with previous studies that showed phenolic aldehydes to be more toxic than phenolic acids on *S. cerevisiae*^19,27–29^. Nonetheless, *Cb*_3974 showed significantly greater capacity to tolerate 4-HBD and syringaldehyde than *Cb*_3904 and *Cb*_wild type, particularly at higher inhibitor concentrations (0.75 and 1.0 g/L; Figs 7–9). This underscores the substrate range of the AKR encoded by *Cbei_3974*. We have previously shown that both AKR and SDR can utilize furfural and HMF (furanic aldehydes) and 4-HBD (phenolic aldehyde) as substrates, with AKR being significantly more active with all three substrates than SDR^1^. The enhanced activity of AKR on 4-HBD and syringaldehyde likely accounts for the greater phenolic aldehyde tolerance of *Cb*_3974 relative to *Cb*_3904 and *Cb*_wild type. Butanol and ABE concentrations were the most affected when *Cb*_3974, *Cb*_3904 and *Cb*_wild type cultures were treated with syringaldehyde. This is because syringaldehyde directly truncates the butanol biosynthesis pathway by inhibiting the activity of coenzyme A transferase (CoAT)—an important enzyme in the butanol pathway^30^. Inhibition of CoAT by syringaldehyde is not mitigated by AKR, thus, butanol concentrations fell considerably in all syringaldehyde-treated cultures, albeit to a greater degree in cultures of *Cb*_3904 and *Cb*_wild type (and much less in cultures of *Cb*_3974).

At the inhibitor concentrations tested, overexpression of *Cbei_*3904 did not confer significantly greater inhibitor tolerance on *Cb*, particularly with furfural. Therefore, we conclude that rapid *in situ* detoxification of inhibitors by the AKR-expressing strain (*Cb*_3974) relieves inhibitor-mediated toxicity, thus leading to a faster rate of and greater final solvent accumulation in cultures of *Cb*_3974. Conversely, whereas likely increases in biotin biosynthesis and/or cell membrane lipid modulation may increase inhibitor tolerance in *Cb*_3904, although marginally, these effects are largely infective on their own at high inhibitor concentrations. This underscores the ability of *Cb* to recruit multifarious mechanisms in response to lignocellulose-derived inhibitors, some of which appear to target *in situ* detoxification while others play protective roles in stabilizing membranes, proteins, and nucleic acids—all of which are directly affected by the inhibitors^16,19,26^. Consequently, some mechanisms are more potent than others. Clearly, *in situ* detoxification to a less toxic compound (such as furfural to furfuryl alcohol) is by far more effective in relieving inhibitor stress than mechanisms that stabilize cellular structures, without removing the toxicant from the growth medium. Engineering a *Cb* strain capable of resisting the combined toxicities of multiple inhibitors in lignocellulosic biomass hydrolysates without significant drop in solvent accumulation, therefore, requires synchronous expression of multiple genes whose protein products target the different toxic effects exerted by LDMICs. However, it is worthy of mention that such an approach should give priority to genes whose protein products are directly involved in inhibitor transformation to less toxic compounds, as demonstrated by the strain, *Cb*_3974 in this study.

## Conclusions

In this study, we engineered and characterized novel *Cb* strains—*Cb*_3974 and *Cb*_3904—by homologous integration for improved detoxification of LDMICs. *Cb*_3974, carrying an additional copy of an aldo-keto reductase encoded by *Cbei_3974* demonstrated enhanced capacity to tolerate furfural, syringaldehyde, and 4-hydroxybenzaldehyde, with considerably higher butanol accumulation relative to *Cb*_3904 and *Cb*_wild type. *Cb*_3974 therefore, represents a promising candidate for enhanced detoxification of furanic and phenolic aldehydes during ABE fermentation of lignocellulosic biomass hydrolysate. Development of *Cb_*3974, which is considerably tolerant to furfural and to a reasonable extent, 4-hydroxybenzaldehyde and syringaldehyde, is a significant step towards fermentation of lignocellulose-derived sugars to butanol. In future studies, we will test the capacity of *Cb*_3974 to ferment lignocellulosic biomass hydrolysates to butanol.

## Materials and Methods

### Declaration of level of biocontainment

All bacteriological work were conducted in compliance with the safety and biocontainment regulations associated with a biological safety level one (BSL-1) laboratory.

### Bacterial strains, plasmids and culture conditions

*C. beijerinckii* NCIMB 8052 (ATCC 51743) used in this study was obtained from the American Type Culture Collection (Manassas, VA). Stocks were stored as spore suspensions in sterile, double-distilled water at 4 °C. *E. coli* TOP 10 cells (New England Biolabs, Ipswich, MA) were used for cloning and maintenance of recombinant plasmids. *E. coli* TOP 10 cells were maintained as glycerol stocks (50% v/v) at −80 °C. The chromosomal integration plasmid, pMTL-JH16 (a generous gift from Dr. Nigel Minton’s laboratory, University of Nottingham, University Park, Nottingham NG7 2RD, United Kingdom) was used for the construction of recombinant plasmids pMTL-JH16_3974 and pMTL-JH16_3904. The list of microorganisms, plasmids and enzymes used in this study are shown in Table 3.

**Table 3:** List of microorganisms, vectors and enzymes used in this study and the respective characteristics and sources.

Strains/vectors/enzymes/primers	Characteristics	Source
***Strains***		
*E. coli* TOP 10 cells	endA1, recA1, gyrA96, relA1	Promega Corporation
*C. beijerinckii* NCIMB 8052_ wild type (*Cb*_ wildtype)	Wildtype	American Type Culture Collection (ATCC)
*C. beijerinckii* NCIMB 8052_3904 (*Cb*_3904)	Erm^r^, SDR	This study
*C. beijerinckii* NCIMB 8052_3974 (*Cb*_3974)	Erm^r^, AKR	This study
***Vectors***		
pMTL-JH16	Erm^r^, Catp^r^	Nottingham University
pMTL-JH16_3904	Erm^r^, Catp^r^, SDR	This study
pMTL-JH16_3974	Erm^r^, Catp^r^, AKR	This study
***Enzymes***		
GXL DNA polymerase	—	Takara Clontech
*Kpn*I	—	New England Biolabs
*Xho*I	—	New England Biolabs
T4 DNA ligase	—	New England Biolabs

To revive *C. beijerinckii* spores, 200 µl of spores were heat-shocked for 10 min at 75 °C, cooled on ice, and then inoculated into 10 ml of anoxic tryptone-glucose-yeast extract (TGY) broth and incubated in an anaerobic chamber (Coy Laboratory Products Inc., Ann Arbor, MI) at 35 °C for 12 to 14 h for inoculum generation as previously described^31^. *E. coli* TOP 10 cells were grown in Luria-Bertani broth. To maintain the recombinant plasmids, *E. coli* TOP 10 carrying the respective recombinant plasmids, pMTL-JH16_3974 and pMTL-JH16_3904, were grown in Luria-Bertani broth supplemented with erythromycin (50 mg/ml) and chloramphenicol (34 mg/ml).

### Cloning of *Cbei_*3974 and *Cbei_*3904

**Figure 11:**
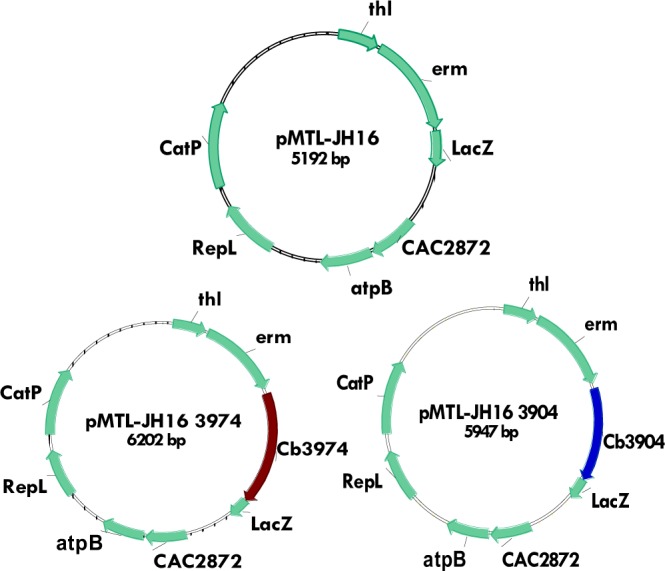
Construction of pMTL-JH16_3974 and pMTL-JH16_3904 from the parent plasmid, pMTL-JH16.

Genomic DNA (gDNA) was extracted from *Cb* as previously described^32^. *Cbei_*3974 and *Cbei_*3904 were amplified by PCR using gDNA as template, specific primers and PrimeStar® GXL DNA polymerase (Clontech-Takara, Mountain View, CA). The primer sequences and PCR strategies used in this study are described in Table 1. PCR was conducted using the following conditions; (1) initial denaturation, 98 °C for 2 min (1 cycle); (2) denaturation, 98 °C for 20 s; annealing, annealing temperature was set between 58–62 °C (depending on the primer; see Table 1 for details) for 30 s, extension, 72 °C for 1 min (35 cycles); (3) final extension, 72 °C for 10 min (1 cycle); (4) hold, 4 °C for 10 min (1 cycle). A 100 µl reaction contained 5X PrimeStar® GXL buffer (20 µl), dNTPs (0.25 mM), primers (0.5 µM each), DNA template (~5 ng/ µl) and GXL DNA polymerase (2 µl). Both constructs (pMTL-JH16_3974 and pMTL-JH16_3904; Fig. 11) were designed to include ribosome binding site (RBS), spacer sequence between the RBS and a start codon upstream of each gene (*Cbei_*3974 and *Cbei_*3904), while a transcription termination sequence was placed downstream of each gene.

**Figure 12:**
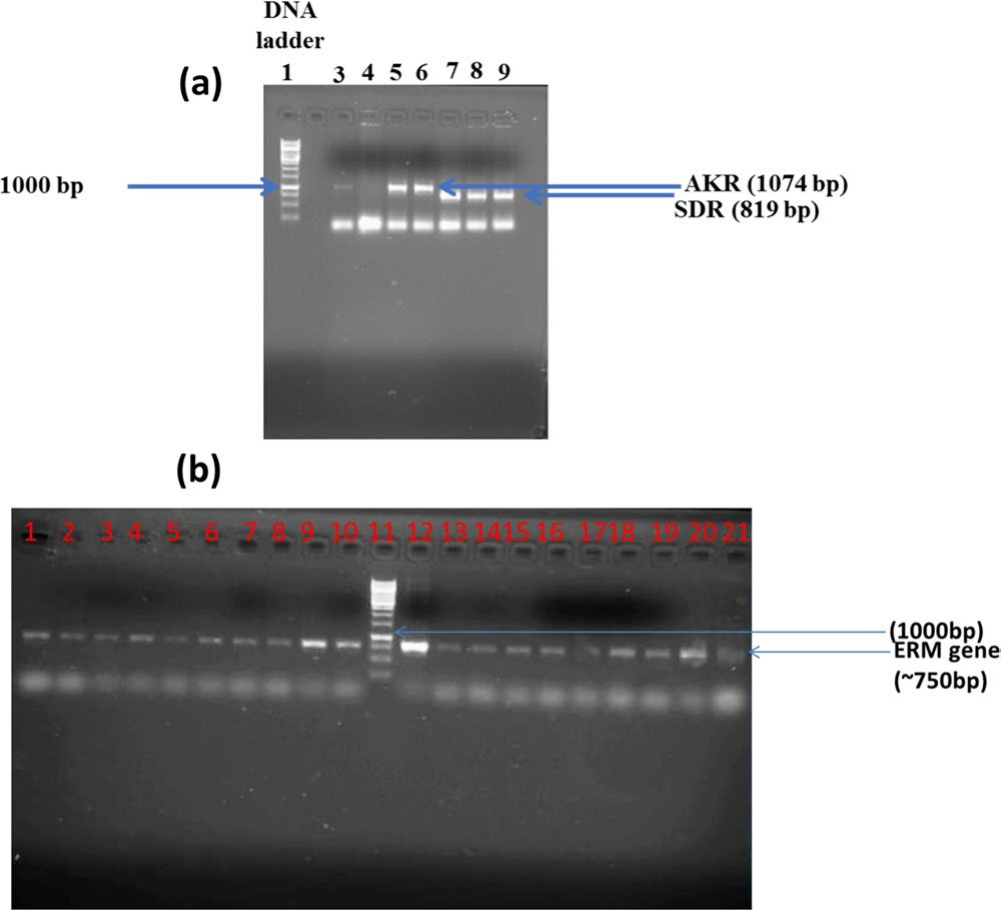
PCR amplification of respective constructs following extraction of recombinant plasmids from respective *E. coli* TOP 10 transformants. (**a**) *AKR* (*Cbei_3974*) and *SDR* (*Cbei_3904*) amplicons following PCR amplification with pMTL-JH16_3974 (lanes 4, 5 and 6) and pMTL-JH16_3904 (lanes 8 and 9) extracted from *E. coli* TOP 10 transformants as template. Lanes 3 and 7: positive controls, where lane 3 is the PCR amplicon using pMTL-JH16_3974 before transformation (into *E. coli*) as template, while lane 7 is the PCR amplicon using pMTL-JH16_3904 before *E. coli* transformation as template. (**b**) Gel image showing PCR amplicons of erythromycin resistance gene using genomic DNA extracted from *Cb* colonies transformed with pMTL-JH16_3904 (lanes 1–10) and pMTL-JH16_3974 (13–21). Lane 12: erythromycin resistance gene positive control. Lane 11: DNA ladder. The gel images were cropped for clarity and the full-length gels are presented in Fig. S18.

The amplicons for *Cbei_*3974 and *Cbei_*3904 were gel-purified following agarose gel electrophoresis using agarose (0.9% w/v) and 1X TAE buffer (40 mM Tris-acetate in 1 mM EDTA, pH 8.0). The purified constructs were digested independently with *Kpn*I and *Xho*I whose recognition sites were included in the respective PCR primers (see Table 1). The integration plasmid pMTL-JH16 was previously used to transform *E. coli* TOP 10 cells and was subsequently extracted using GenCatch plasmid DNA miniprep kit (Epoch Life Science, Sugar Land, TX). The extracted pMTL-JH16 plasmid was digested with *Kpn*I and *Xho*I. The digested *Cbei_*3974, *Cbei_*3904 and pMTL-JH16 were subsequently gel-purified using the GenCatch advanced PCR extraction kit (Epoch Life Science, Sugar Land, TX). The digested *C. bei_*3974 and *Cbei_*3904 were separately ligated into the predigested pMTL-JH16 using an insert to vector ratio of 10:1. Overnight ligation reaction was performed at 16 °C using T4 DNA ligase (New England Biolabs, Ipswich, MA). Following ligation, T4 DNA ligase was heat-inactivated at 65 °C for 10 minutes and the ligation mixtures were column-purified using GenCatch advanced PCR extraction kit (Epoch Life Science, Sugar Land, T X). The ligation products were used to transform competent *E. coli* TOP 10 cells by heat-shocking at 42 °C for 60 s. Prior to heat-shocking, 2 µl of the ligation mixture was mixed with 40 µl competent *E. coli* TOP 10 cells and the mixture was placed on ice for 5 minutes before heat-shocking. The heat-shocked mixtures were placed on ice for 5 minutes and then, the cells were diluted using 250 µl SOC medium (2% w/v bacto-tryptone, 0.5% w/v yeast extract, 10 mM NaCl, 2.5 mM KCl, 10 mM MgCl_2_, and 20 mM glucose), followed by incubation for 1 h at 37 °C and 250 rpm. The recovered cells were plated on Luria-Bertani agar with the addition of erythromycin (100 µg/ml) followed by incubation for 12–16 h until distinct colonies were observed. Colonies were picked from Luria-Bertani agar plates and subsequently cultured in Luria-Bertani broth with addition of 7.5 µg/ml chloramphenicol. The resulting *E. coli* cells for each construct were harvested and the recombinant plasmids extracted using GenCatch plus plasmid DNA miniprep kit (Epoch Life Science, Sugar Land, TX). PCR was conducted to screen for the presence of *Cbei_*3974 and *Cbei_*3904 using the extracted plasmids as templates. PCR confirmed presence of *Cbei_*3974 and *Cbei_*3904 in the respective colonies (Fig. 12a). The *E. coli* colonies harboring recombinant pMTL-JH16_ 3974 and pMTL-JH16_3904 were cultured in large volumes and the recombinant plasmids were extracted using Fast Ion Plasmid Midi Kit (IBI Scientific, Peosta, IA).

### Homologous recombination of *Cbei_*3974 and *Cbei_*3904 in *C. beijerinckii*

To facilitate chromosomal integration of *Cbei_*3974 and *Cbei_*3904 in *Cb*, we utilized pMTL-JH16 plasmid designed for heterologous gene integration in *Clostridium* species. The plasmid, pMTL-JH16 contains 300 bp of thiolase (*thl*) promoter and 1200 bp (*CA_C*2872/*atpB*) downstream of *thl* promoter sequence of *Clostridium acetobutylicum* to enable double-cross homologous recombination. *CA_C*2872 and *atpB* code for a membrane protein and an ATP synthase F_0_/F_1_ subunit A in *C*. *acetobutylicum*, respectively. *Cbei_*3974 and *Cbei_*3904 were inserted into pMTL-JH16 multiple cloning sites between the *thl* promoter/*erm* gene and *CA_C*2872/*atpB* as shown in Fig. 11.

The extracted pMTL-JH16_ 3974 and pMTL-JH16_3904 were used to transform electro-competent *C. beijerinckii*. Competent *Cb* cells were prepared by harvesting *Cb* at 2500 rpm and 4 °C for 10 minutes from pre-culture grown overnight (under anaerobic condition; 80% nitrogen, 16.5% CO_2_ and 3.5% hydrogen) to an optical density (OD_600_) of 0.7. The cells were washed twice with 10% PEG-8000 (polyethylene glycol 8000) at 2500 rpm and 4 °C. The resulting competent cells were re-suspended in 2 ml 10% PEG-8000. Competent *Cb* cells were transformed with recombinant pMTL-JH16_ 3974 and pMTL-JH16_3904, respectively by electroporation in a 2-mm cuvette using Bio-Rad Gene Pulser Xcell™ (BioRad, Hercules, CA) set at a voltage of 2.5 KV with a capacitance of 25 µF and infinite resistance (∞ Ω). The electroporation time ranged from 4.1 to 5.6 ms. Following electroporation, cells were diluted in 3 ml TGY medium and incubated for 8 h at 35 °C. The recovered cells were plated on TGY agar supplemented with erythromycin (25 µg/ml) followed by incubation at 35 °C for 24–48 h until discrete colonies were visible. Colonies were picked and re-plated on TGY agar with addition of 25 µg/ml erythromycin. Subsequently, colonies were picked and inoculated into TGY broth with addition of erythromycin (25 µg/ml).

Cells were harvested from cultures of *Cb*_3974 and _3904 grown in TGY broth and genomic DNA was extracted from each strain. PCR was conducted to screen for the presence of erythromycin resistance gene in each strain (*Cb*_3974 and _3904) using genomic DNA as template. Erythromycin resistance gene in the genome of *Cb* was targeted to confirm presence of each construct including both of the target genes (*Cbei_*3974 and *Cbei_*3904) inserted into *Cb* genome. Figure 12b shows the presence of erythromycin resistance gene in both recombinant strains (*Cb*_3974 and _3904). To further confirm genomic integration of *Cbei_3974* and *Cbei_3904* in *Cb*, plasmid curing was conducted followed by replica plating as described previously^33^. Presence of the flanking regions of the integration constructs (*thl* and *CA_C*2872/*atpB*) were also confirmed by PCR. *Cb_*3974 and *Cb_*3904 were plasmid-cured using mitomycin-C. Briefly, *Cb_*3974 and *Cb_*3904 strains were inoculated independently into TGY medium supplemented with 120, 240, 360 and 720 ng/ml mitomycin-C and incubated for 36 h under anaerobic condition. Cultures of *Cb_*3974 and *Cb_*3904 containing 360 ng/ml mitomycin-C (the highest mitomycin-C concentration tolerated by the cells) that showed observable growth were plated on TGY agar without erythromycin supplementation and incubated at 35 °C overnight. Colonies from *Cb_*3974 and *Cb_*3904 on TGY agar plates were picked and re-plated on fresh TGY agar plates supplemented with 25 µg/ml erythromycin and incubated overnight. Colonies were observed on TGY agar plates supplemented with 25 µg/ml erythromycin, hence confirming successful genomic integration (Fig. S15). Further, colonies from replica TGY agar plates for both *Cb_*3974 and *Cb_*3904 were inoculated into TGY broth supplemented with 25 µg/ml erythromycin and incubated for 24 h. Cells for both strains were then harvested and gDNA was extracted. PCR was conducted in two steps using gDNA from Cb_wild type and plasmid-cured *Cb_*3974 and *Cb_*3904 as templates. In the first step, the thiolase-*Cbei_3974*/*Cbei_3904*-erythromycin regions of the construct were amplified with the primer pairs Thl_pMTL-JH16 forward and Cb_3974/Cb_3904 reverse1 (Table 1). In the second step, *Cb-3974*/*Cb_3904* forward and *atpB* reverse primers were used to amplify the *Cbei*_*3974*/*Cbei_3904*-*CA_C*2872/*atpB* regions. CatP forward and CatP reverse primers were used to amplify the *CatP* gene (Table 1). The plasmids, pMTL-JH16_ 3974, pMTL-JH16_3904 and pMTL-JH16 were used as positive controls for the PCR amplifications described above. Gel image results presented in Fig. 2 confirm the absence of plasmids in both recombinant *Cb* strains, Cb_3974 and Cb_3904 (Fig. 2a), and the presence of both chromosomally integrated genes and their flanking regions: *thl*-*erm*-*Cbei*_3974-*CA_C*2872/*atpB* for *Cb*_3974 and *thl*-*erm*-*Cbei*_3904-*CA_C*2872/*atpB* for *Cb*_3904 (Fig. 2b,c).

### Adaptation of *Cb*_3974 and _3904

Following genomic integration of *Cbei_*3904 and *_*3974 in *Cb*, the generated recombinant strains (*Cb*_3904 and _3974) grew poorly. In an effort to increase their growth profile, both strains were adapted to erythromycin. Colonies were picked from agar plate cultures of *Cb*_3974 and _3904 and transferred into TGY broth supplemented with 25 µg/ml erythromycin. The cultures were incubated at 35 °C for 12–60 h. At first, growth was observed after 48 h of incubation for *Cb*_3904, whereas growth was observed for *Cb*_3974 after 60 h. Subsequently, both strains (*Cb*_3974 and _3904) were sub-cultured (100 µl each) into 5 ml of sterile pre-anoxic TGY broth supplemented with 25 µg/ml erythromycin. The sub-culturing procedure was repeated three times until significant growth was observed for *Cb*_3904 and _3974 in 14 h and 24 h, respectively. Consequently, *Cb*_3904 and _3974 were cultured in P2 medium with the addition of 25 µg/ml erythromycin and grown at 35 °C for 2 weeks, which allows for sporulation and settling of the spores to the bottom of culture broth. After 2 weeks, the spores were harvested by centrifugation at 3000 × g for 10 minutes. The spores were washed 8–10 times with sterile double-distilled water following each centrifugation step. The spores were then stored as a suspension in sterile double-distilled water at 4 °C.

### Assessing the stability of *Cb*_3974 and _3904

The stability of *Cb*_3974 and _3904 were tested during acetone-butanol-ethanol fermentation. First, the generation times of *Cb*_3974 and _3904 were determined by growing both strains in P2 medium containing glucose without antibiotics. Samples were collected at intervals and analyzed for optical density (OD_600 nm_) using DU® 800 spectrophotometer (Beckman Coulter Inc., Brea, CA) until the cultures entered stationary growth phase. The generation time for each clone was determined from the exponential phase of the growth curves (see supplementary material; Figs S16 and S17). Subsequently, the pre-culture for each strain was prepared and transferred into fresh sterile P2 medium containing glucose without antibiotic supplementation, and the cultures were grown for 50 generations following multiple transfers. For instance, for *Cb*_3904, the culture was transferred to fresh P2 medium after 9 h of growth to attain 3 generations (i.e. generation 3, G_3_). Then, the cultures from generation 3 were grown for another 9 h and transferred to fresh P2 medium to attain generation 6, and this was repeated until generation 50 was attained. Additionally, ABE fermentations were conducted in triplicates using the cultures from generations 0, 10, 20, 30, 40, and 50 (G_0_–G_50_), and samples were taken and analyzed for growth and ABE production. Further, cultures from G_0_, G_10_, G_20_, G_30_, G_40_ and G_50_ were plated on sterile TGY agar without erythromycin supplementation (Fig. S8). The resulting colonies were then transferred to fresh TGY broth without erythromycin supplementation and grown to an OD of 0.5 (OD_600 nm_). Afterwards, the cells were harvested, and genomic DNA was extracted using standard procedure. PCR was conducted using the genomic DNA from the different generations as template to screen for the presence of erythromycin resistance gene. pMTL-JH16 plasmid was used as a positive control (Fig. S7). In addition, the colonies from TGY agar plates without erythromycin supplementation were suspended in sterile TGY broth and then plated on fresh sterile TGY agar supplemented with erythromycin (20 µg/ml). The plates were incubated for 12–36 after which the appearance of colonies was observed (Fig. S9). Additionally, *Cb*_3974 and _3904 were assessed for ABE production up to generation 50.

### Characterization of *Cb*_3974 and _3904

To evaluate *Cb*_3974 and _3904 for growth and LB-derived inhibitor detoxification and tolerance, butanol fermentation in P2 medium was conducted with or without addition of LB-derived inhibitors. The LB-derived inhibitors tested include furfural, vanillic acid, syringic acid, syringaldehyde, and 4-hydroxybenzaldehyde. Furfural tolerance was tested at 4, 5 and 6 g/L, pulse-fed to the fermentation medium when cell optical density (OD_600_) reached ~2. Tolerance to each phenolic compound was tested at 0.5, 0.75 and 1.0 g/L (vanillic acid, ferulic acid, syringic acid, syringaldehyde, and 4-hydroxybenzaldehyde). Phenolic compounds were added to P2 fermentation medium at 0 h of fermentation. Spore suspensions (200 μl) of wildtype *Cb, Cb*_3974 and _3904 were heat-shocked at 75 °C for 10 min followed by cooling on ice. The individual spores were transferred into 10 ml pre-anoxic sterile TGY supplemented with erythromycin (20 µg/ml) and incubated at 35 °C for 12–24 h to obtain the pre-culture. The pre-culture was transferred to 90 ml of TGY when cell optical density reached 0.9–1.1 and incubated further at 35 °C for 3–4 h until optical density reached 0.9–1.1. The pre-culture was then used to inoculate the fermentation medium. Fermentation was conducted in loosely-capped 100 mL Pyrex culture bottles at 35 ± 1 °C using 6% (v/v) of the preculture as inoculum in P2 medium^31,34^. The P2 fermentation medium was buffered with 2-(N-morpholino) ethanesulfonic acid (MES; 7 g/L). Fermentation was conducted in triplicate in an anaerobic chamber (Coy Laboratory Products Inc., Ann Arbor, Michigan), with a modified atmosphere of 82% N_2_, 15% CO_2_, and 3% H_2_.

### Real-time quantitative reverse transcriptase polymerase chain reaction (qRT-PCR)

To determine the expression levels of the homologously integrated genes, the messenger RNA (mRNA) levels of *Cbei_*3904 (SDR) and *Cbei_*3974 (AKR) genes in *Cb* clones were quantified. Specific primers for *Cbei_*3904 and *Cbei_*3974 were used for the quantitative real-time PCR (qRT-PCR). *Cb*_WT, *Cb*_3904 (SDR) and *Cb*_3974 (AKR) were grown in P2 medium and cells harvested at 12 h of growth. Total RNA was isolated with Tri Reagent® (Sigma, St. Louis, MO) using cell pellets from 4-ml culture aliquots. Cell pellets were obtained after centrifugation at 10000 × g for 10 minutes. The pellets were then resuspended in 1 ml of Tri Reagent® and disrupted in a Tissue Lyser LT (Qiagen, Hilden Germany) at 50 oscillations/s for 2 min. The RNA purity was determined spectrophotometrically using NanoDrop (BioTek® Instrument Inc. Winooski, VT). Only RNA samples with 260/280 ratios ≥2.0 were used for qRT-PCR. Total RNA (2 µg) was reverse transcribed into complementary DNA (cDNA) using Random Hexamers (Qiagen, Hilden Germany) and M-MLV Reverse Transcriptase (Promega, Madison, WI) according to the manufacturer’s protocol. Then, qRT-PCR was performed in triplicates using the cDNA as templates and GoTag® qPCR Master Mix containing BRYT Green® (Promega, Madison, WI) in a Bio-Rad CFX96 Touch Deep Well™ Real-Time Detection Systems (Bio-Rad, Hercules, CA). The qRT-PCR conditions were: 1 cycle of 95 °C at 15 min (initial denaturation), 40 cycles of 95 °C at 10 s (denaturation), and 55 °C at 60 s (annealing and extension) followed by a melting curve analysis conducted via temperature ramp from 55 °C to 95 °C with 1 °C per 10 s. The mRNA expression levels of *Cb_*3904 and *Cb_*3974 genes were normalized to the 16S rRNA (internal standard) and relative expression was performed by the comparative 2^−ΔΔCT^ method as previously described^35^.

### Analytical methods

Cell growth was determined by measuring optical density at 600 nm (OD_600_) using a DU® 800 spectrophotometer (Beckman Coulter Inc., Brea, CA). Acetone, butanol and ethanol concentrations were quantified from culture supernatants by gas chromatography using 7890A system (Agilent Technologies 7890, Agilent Technologies Inc., Wilmington, DE, USA), equipped with a flame ionization detector (FID) and a J × W 19091 N-213 capillary column [30 m (length), 320 µm (internal diameter) and 0.50 µm (HP-Innowax film thickness)]. Nitrogen was used as the carrier gas, and the inlet and detector temperatures were maintained at 250 and 300 °C, respectively. The oven temperature was set to span from 60 to 200 °C with increments of 20 °C/min, and a 5-min hold at 200 °C. One microliter of each sample was injected into the gas chromatography with a split ratio of 10:1. Residual glucose was analyzed using a Waters 2796 Bioseparations HPLC Module (Waters, Milford, MA, USA) equipped with evaporative light scattering detector (Waters, Milford, MA, USA) and a 9-mm Aminex HPX-87P, 300 mm (length) × 7.8 mm (internal diameter) column and a 30 mm (length) × 4.6 mm (internal diameter) Aminex deashing guard column (Bio-Rad, Hercules, CA, USA). The analysis was performed at 65 °C using HPLC-grade water as the mobile phase, operated at a flow rate of 0.6 mL/min. Residual furfural and furfuryl alcohol concentrations were determined by measuring maximum absorption at 275 and 220 nm, respectively, using a DU® 800 spectrophotometer. The concentrations of furfural and furfuryl alcohol were confirmed by HPLC equipped with a photodiode array detector (Waters, Milford, MA, USA) and a 3.5 µm Xbridge C18, 150 mm (length) × 4.6 mm (internal diameter) column (Waters, Milford, MA, USA) as described previously^36,37^. ABE yield and productivity were calculated as total grams of ABE produced per total grams of glucose utilized and total concentration (g/L) of ABE divided by fermentation time (h), respectively^38^.

## Supplementary information

Supplementary Materials
