# Supplementary material for: Chromosomal integration of aldo-keto-reductase and short-chain dehydrogenase/reductase genes in Clostridium beijerinckii NCIMB 8052 enhanced tolerance to lignocellulose-derived microbial inhibitory compounds
Source: Sci Rep. 2019 May 21;9:7634. doi: 10.1038/s41598-019-44061-1 (PMC6529405; doi:10.1038/s41598-019-44061-1)
Supplement: Supplementary file 1 — Supplementary Materials [file 41598_2019_44061_MOESM1_ESM.pdf]

Chromosomal integration of aldo-keto-reductase and short-chain dehydrogenase/reductase genes in *Clostridium beijerinckii* NCIMB 8052 enhanced tolerance to lignocellulose-derived microbial inhibitory compounds

Running Title: Metabolic engineering of *Clostridium beijerinckii*

Christopher Chukwudi Okonkwo[1], Victor Ujor[2], and Thaddeus Chukwuemeka Ezeji[1*]

1: Department of Animal Sciences, The Ohio State University, and Ohio State Agricultural Research and Development Center (OARDC), 305 Gerlaugh Hall, 1680 Madison Avenue, Wooster, OH 44691, USA

2: Bioenergy and Biological Waste Management Program, Agricultural Technical Institute, The Ohio State University, 1328 Dover Road, Wooster OH 44691, USA

*To whom correspondence should be addressed: Department of Animal Sciences, The Ohio State University, and Ohio State Agricultural Research and Development Center (OARDC), 305 Gerlaugh Hall, 1680 Madison Avenue, Wooster, OH 44691, USA

E-mails: Ezeji.1@osu.edu; Phone: 330-263-3796; Fax: 330-263-3949.

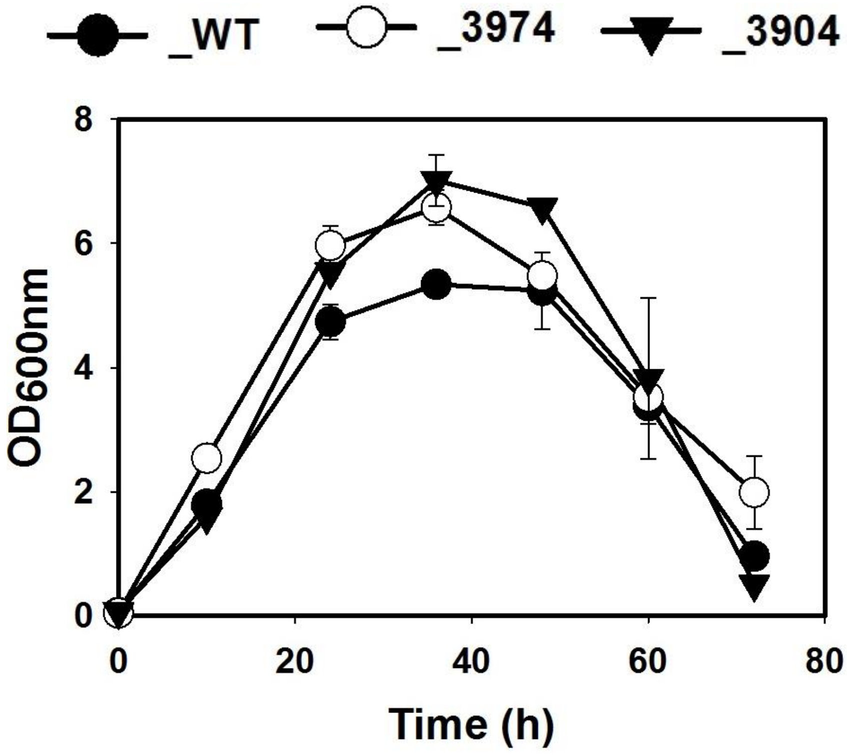

Figure S1. The growth profiles of *Cb*_3974, *Cb*_3904, and *Cb*_wild type in P2 fermentation medium.

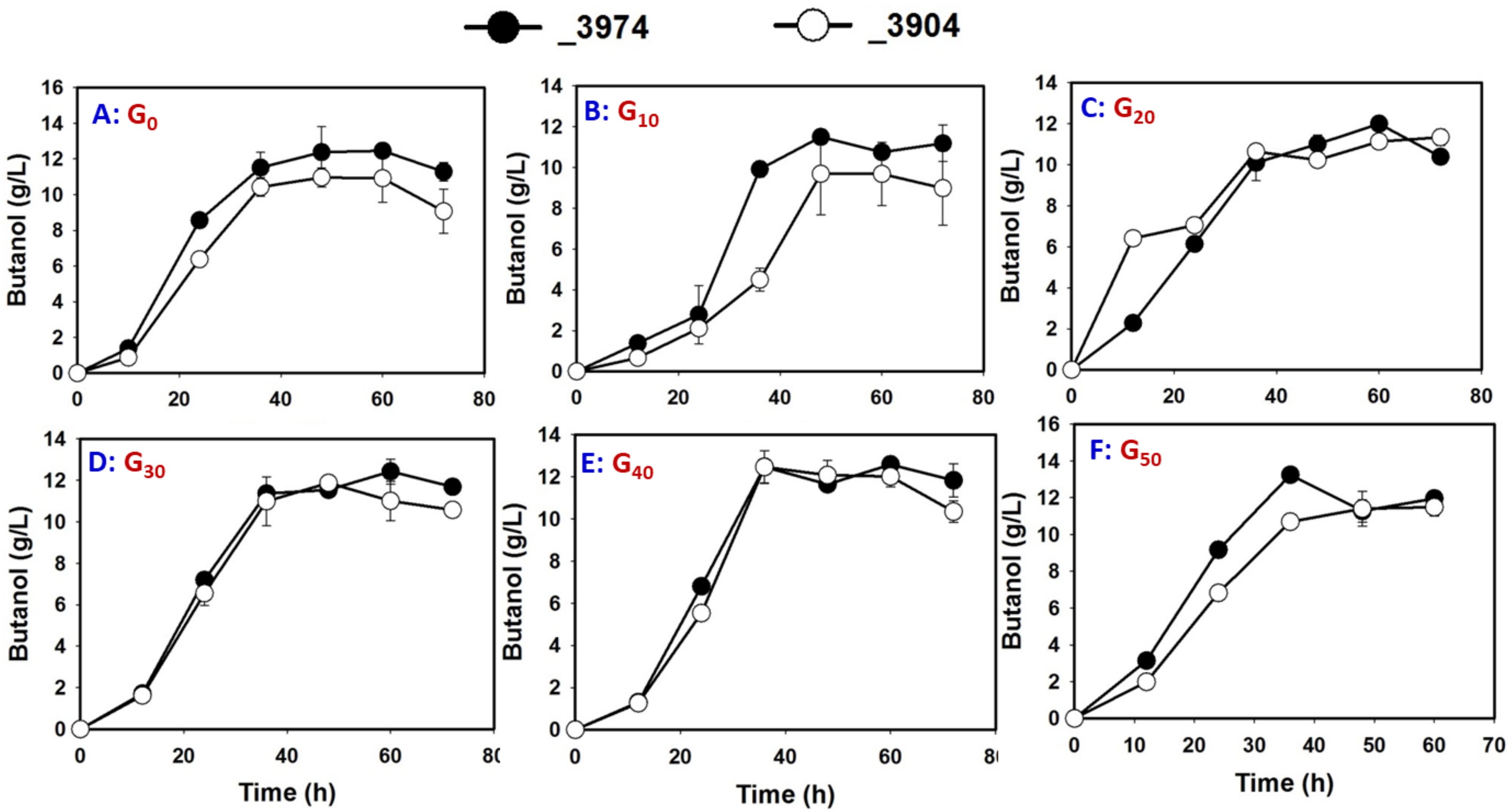

Figure S2. Butanol production by *Cb*_3904 and _3974 over 50 generations.

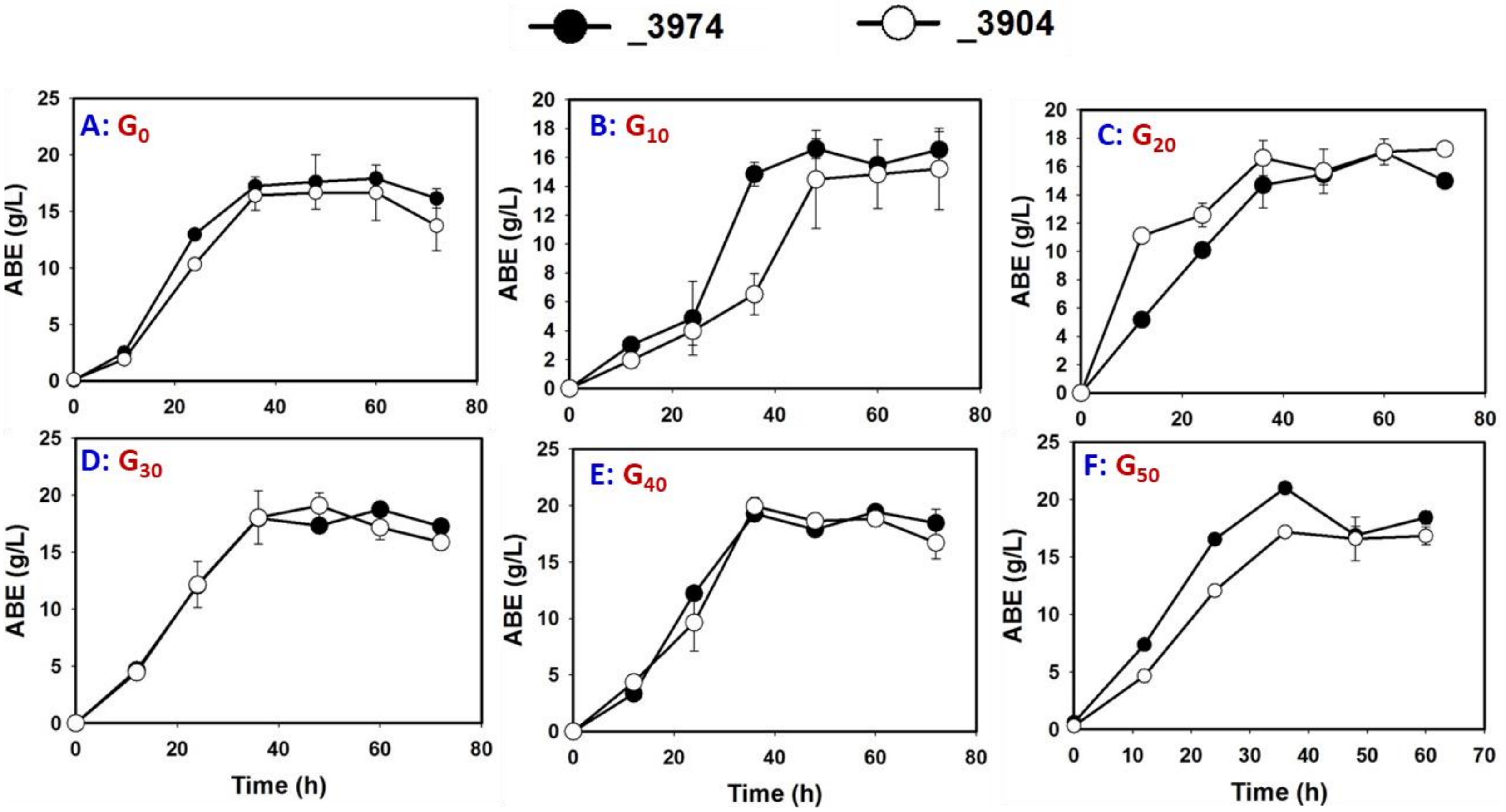

Figure S3. ABE production by *Cb*_3904 and _3974 over 50 generations.

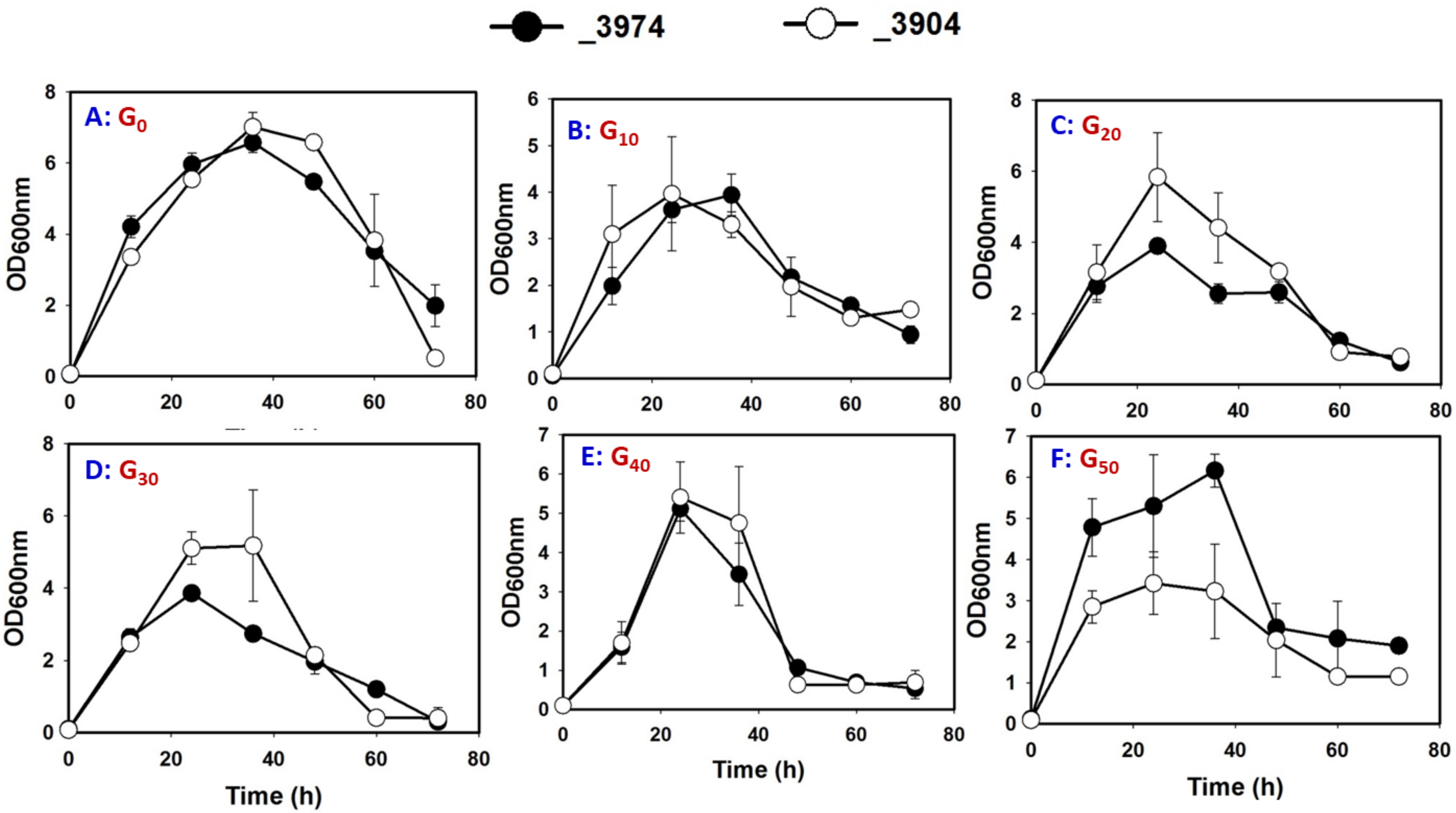

Figure **S4**. The optical densities of *Cb*_3904 and _3974 over 50 generations.

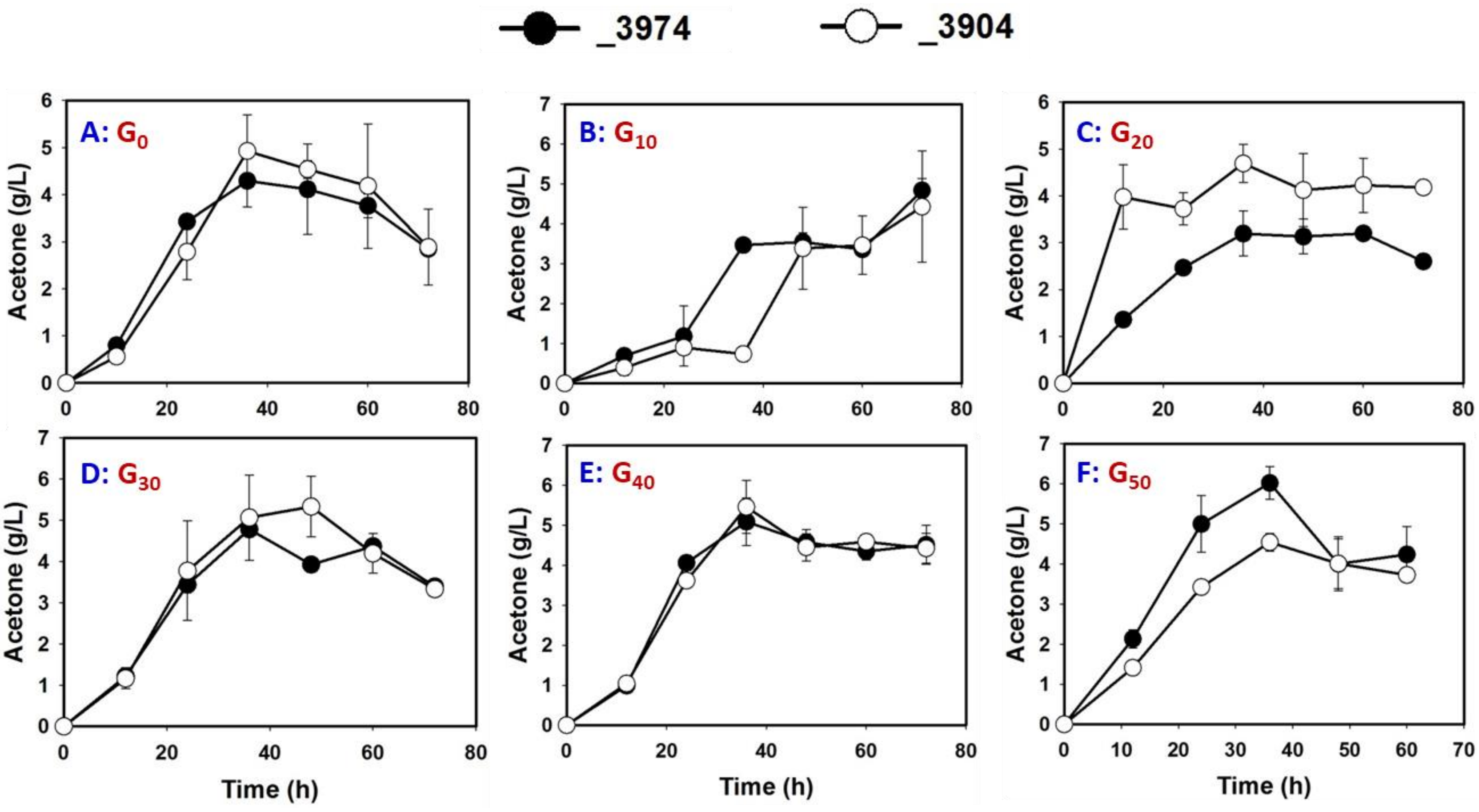

Figure S5. Acetone production profile by *Cb*_3904 and _3974 over 50 generations.

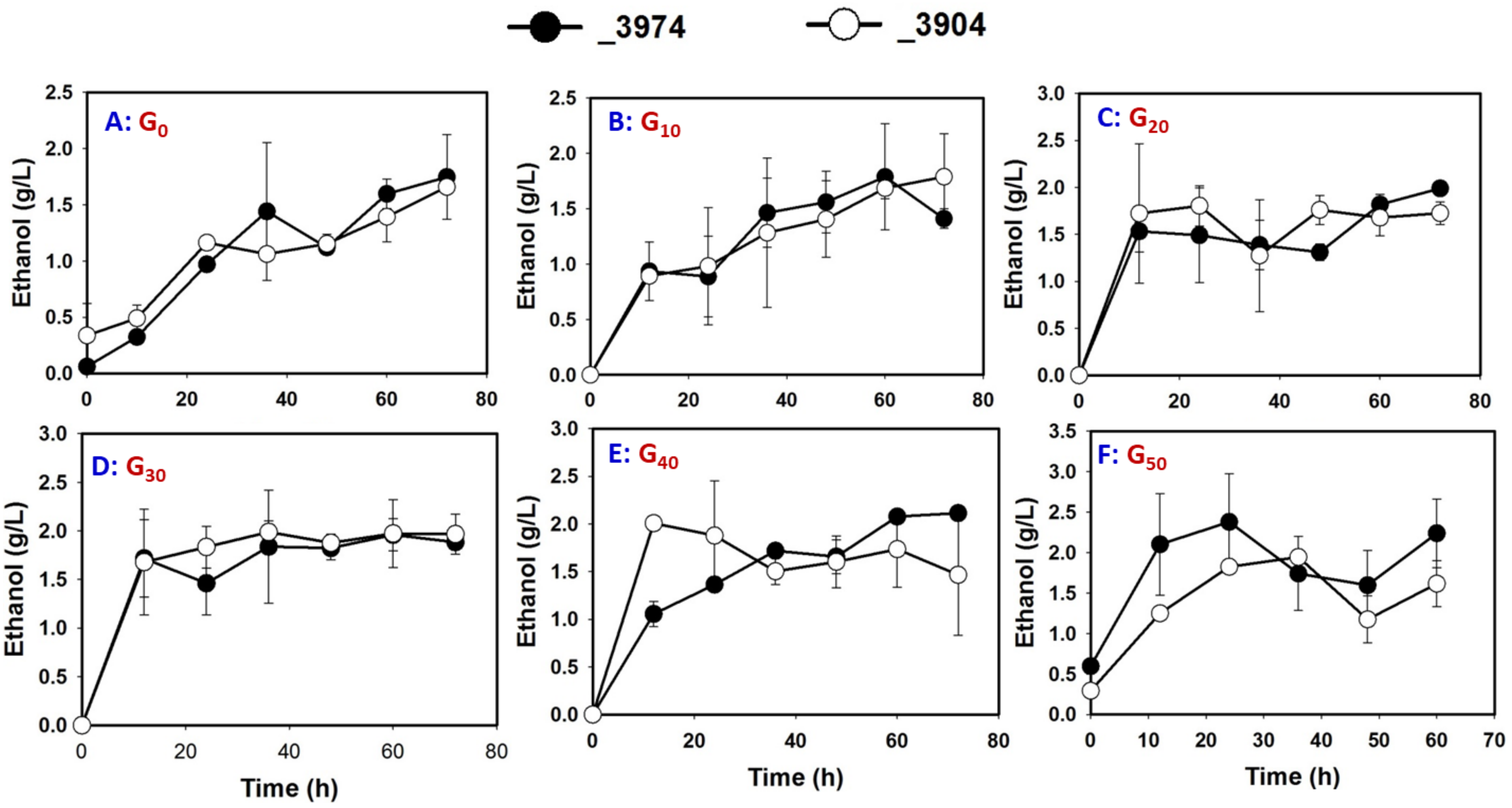

Figure S6. Ethanol production profile by *Cb*_3904 and _3974 over 50 generations.

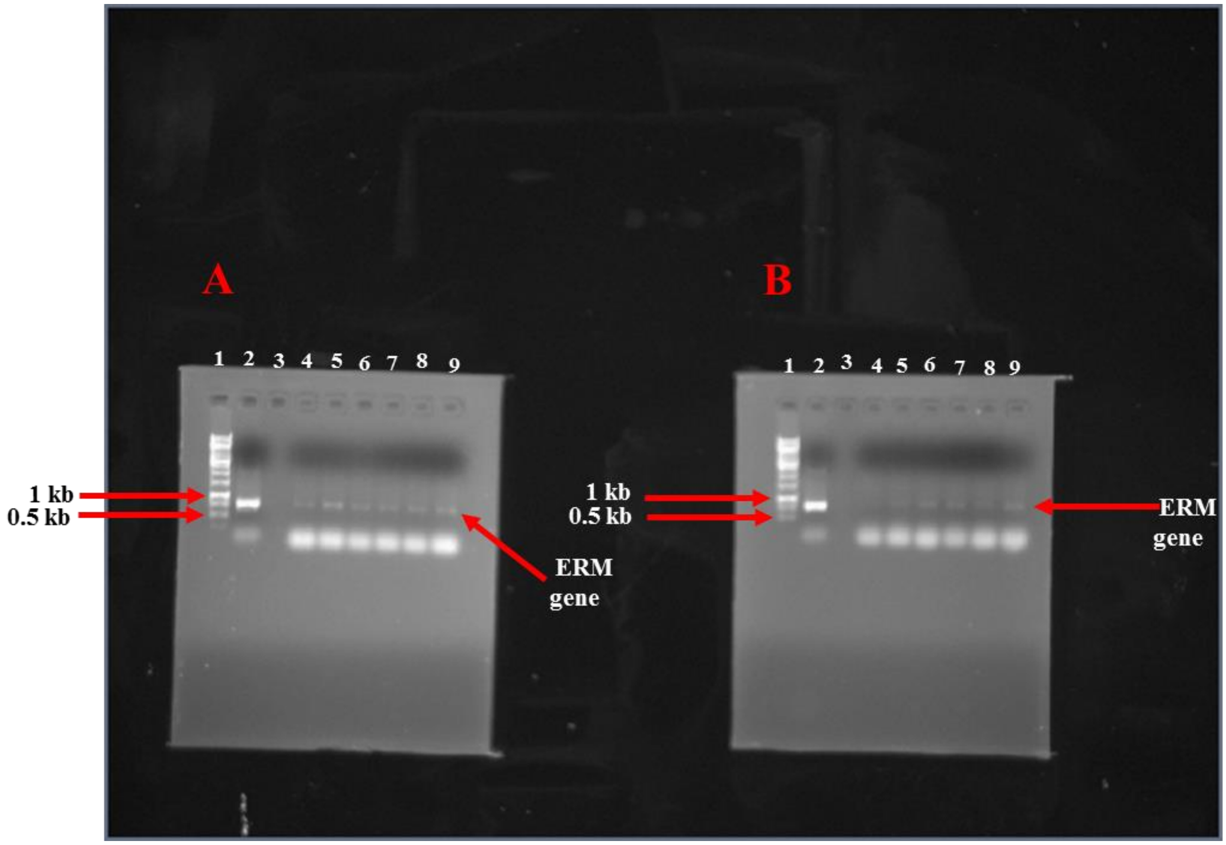

Figure S7. Gel image showing the PCR products from different generations of *Cb*_3974 (**A**: AKR) and *Cb*_3904 (**B**: SDR). Lanes 1 and 2 in **A** and **B** represent 1kb DNA ladder and erythromycin (*erm*) gene control (from pMTL-JH16 plasmid containing erythromycin resistance gene), respectively. Lanes 4, 5, 6, 7, 8 and 9 in **A** and **B** represent generations 0, 10, 20, 30, 40, and 50 of *Cb*_3974 and *Cb*_3904, respectively.

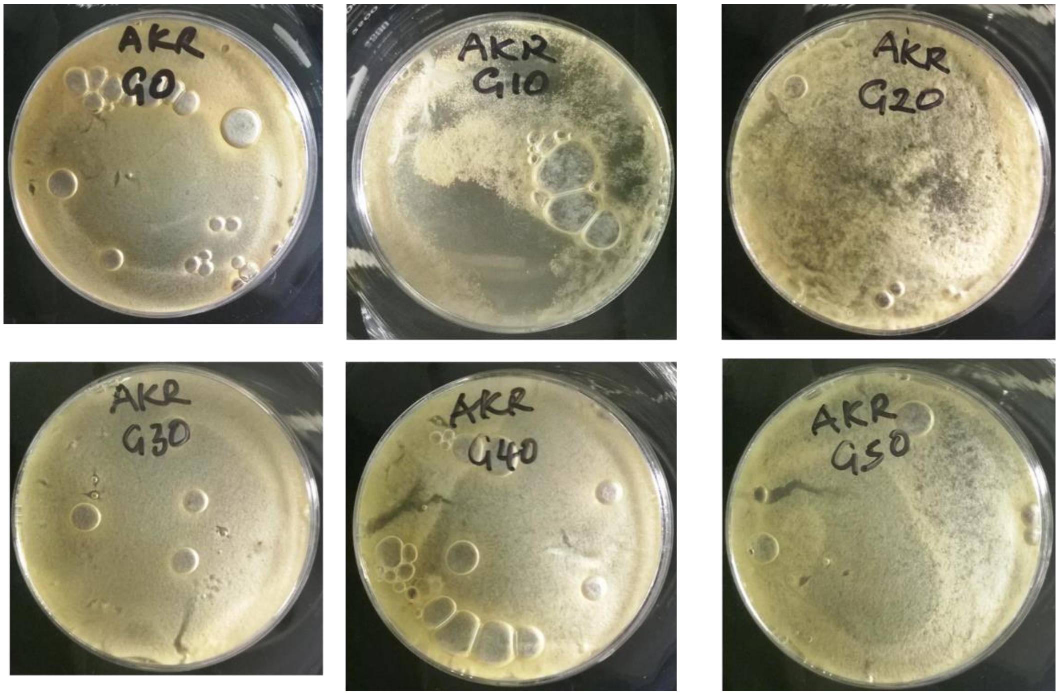

Figure S8A. *Cb*_3974 (AKR) from generations 0, 10, 20, 30, 40 and 50 plated on TGY agar without erythromycin supplementation.

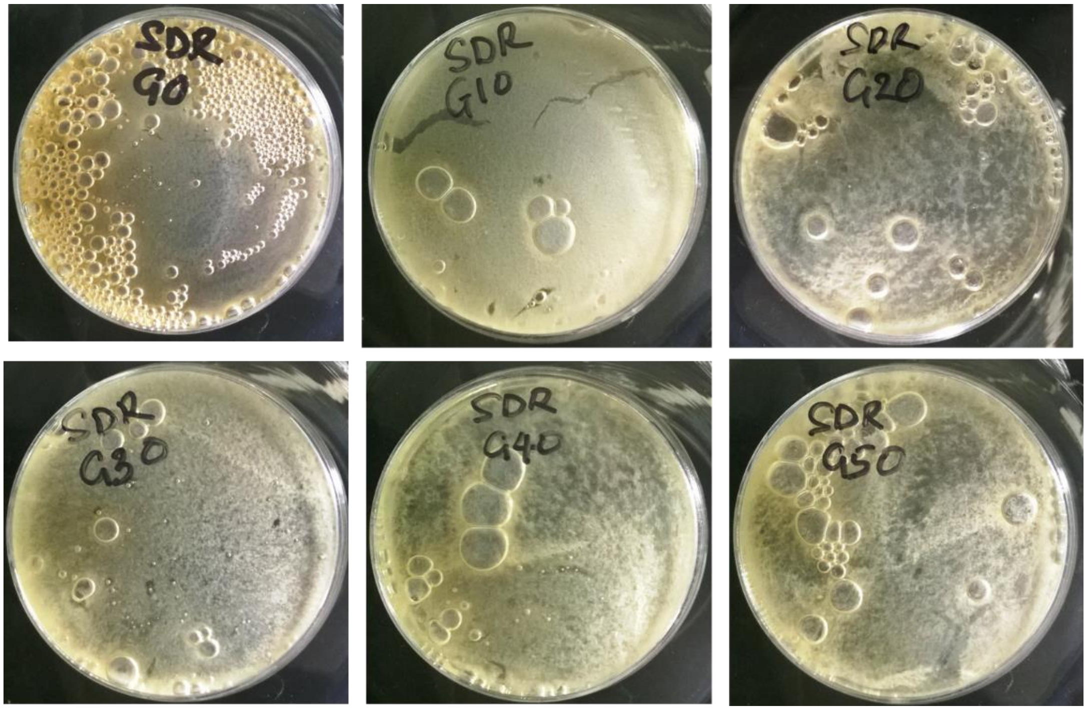

Figure S8B. *Cb*_3904 (SDR) from generations 0, 10, 20, 30, 40 and 50 plated on TGY agar without erythromycin supplementation.

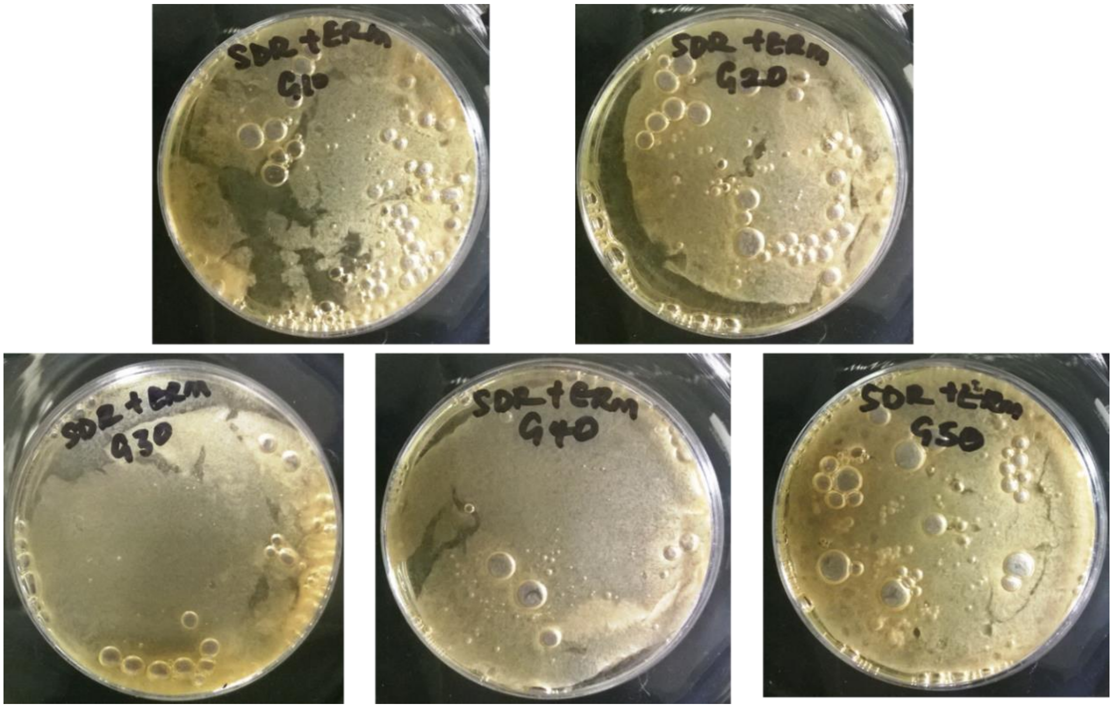

Figure S9A. *Cb*_3974 (AKR) from generations 0, 10, 20, 30, 40 and 50 plated on TGY agar with erythromycin supplementation.

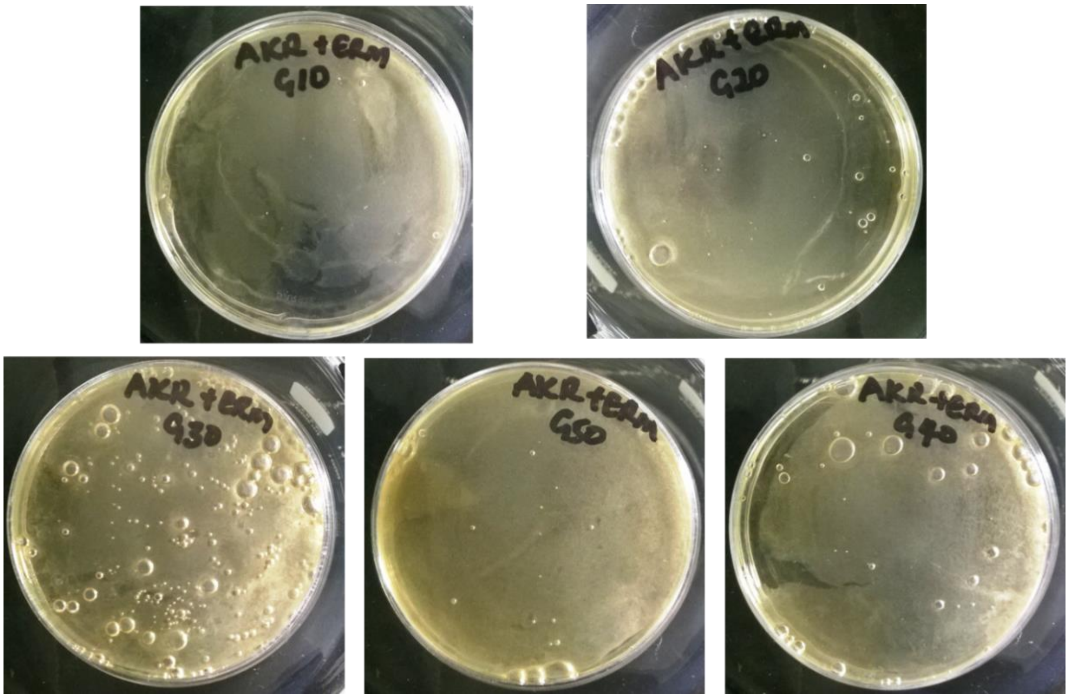

Figure S9B. *Cb*_3904 (SDR) from generations 0, 10, 20, 30, 40 and 50 plated on TGY agar with erythromycin supplementation.

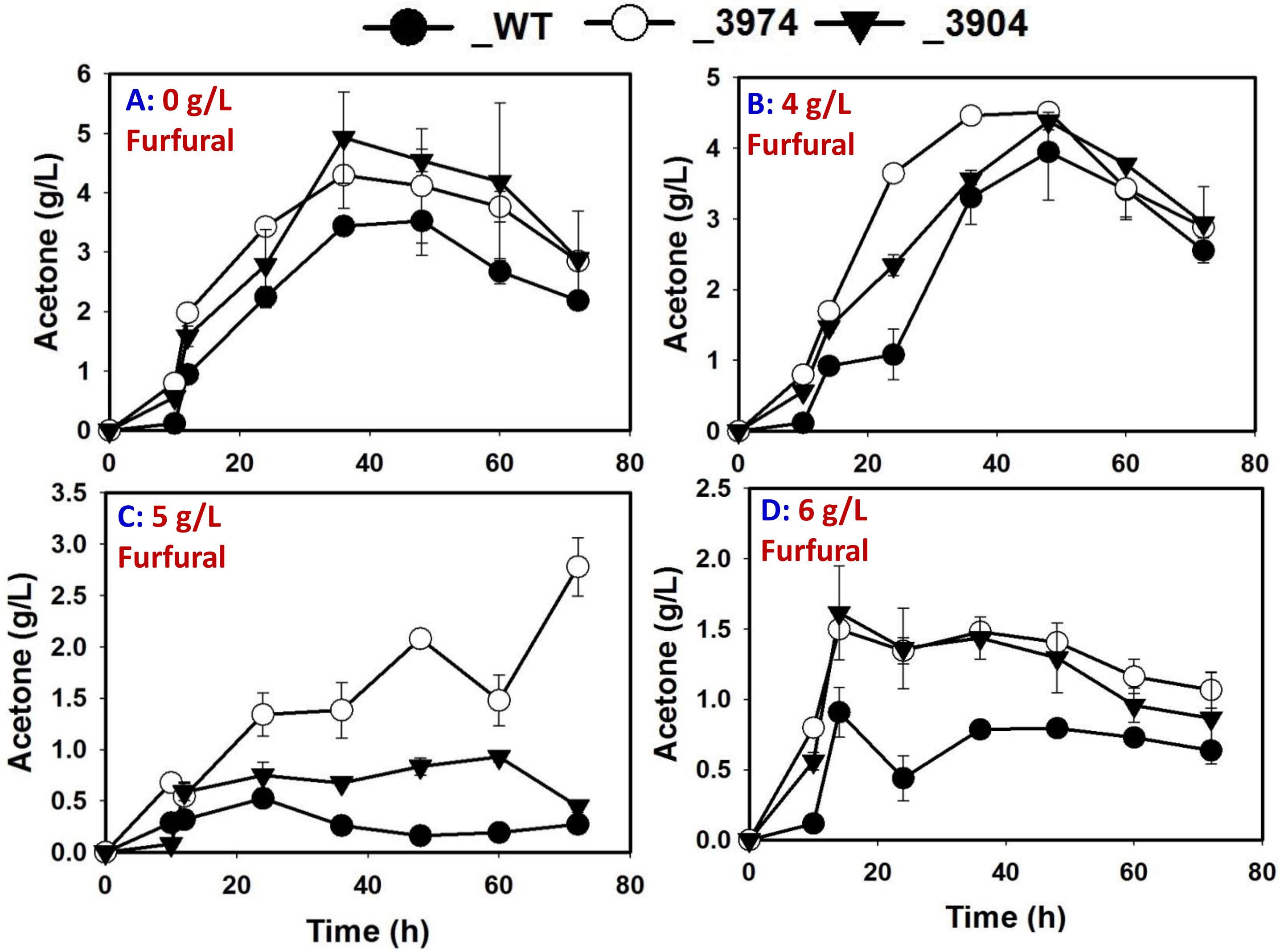

Figure S10. Acetone production profiles of *Cb*_3904, *Cb*_3974, and *Cb*_wild type in cultures supplemented with 0 – 6 g/L furfural

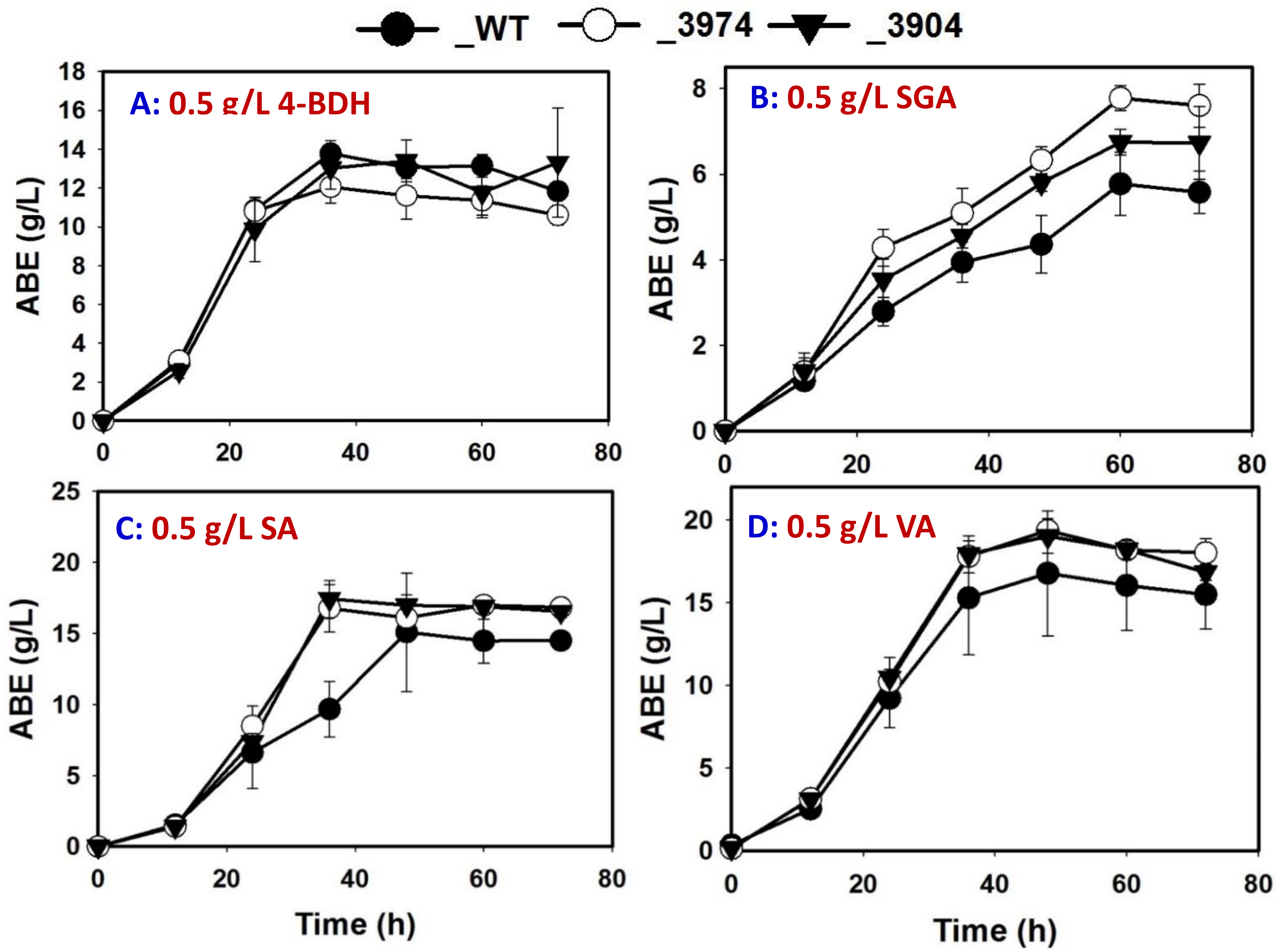

Figure S11. ABE concentrations produced by *Cb*_3974, *Cb*_3904, and *Cb*_wild type in fermentation cultures supplemented with 0.5 g/L 4-hydroxybenzaldehyde (4-HBD), syringaldehyde (SGA), syringic acid (SA), and vanillic acid (VA).

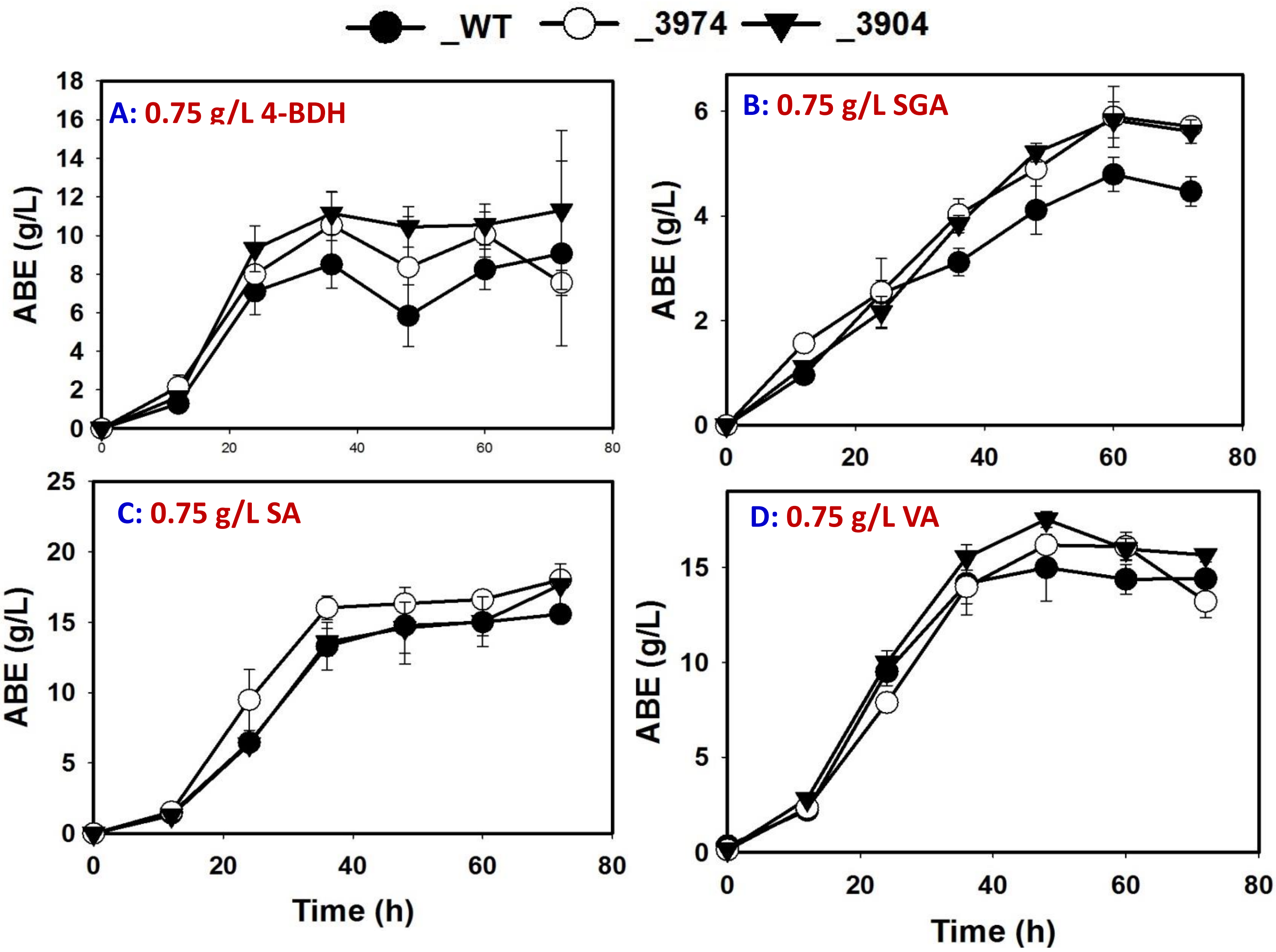

Figure S12. ABE profiles of *Cb*_3974, *Cb*_3904, and *Cb*_wild type during fermentations supplemented with 4-hydroxybenzaldehyde (4-HBD), syringaldehyde (SGA), syringic acid (SA), and vanillic acid (VA; 0.75 g/L each).

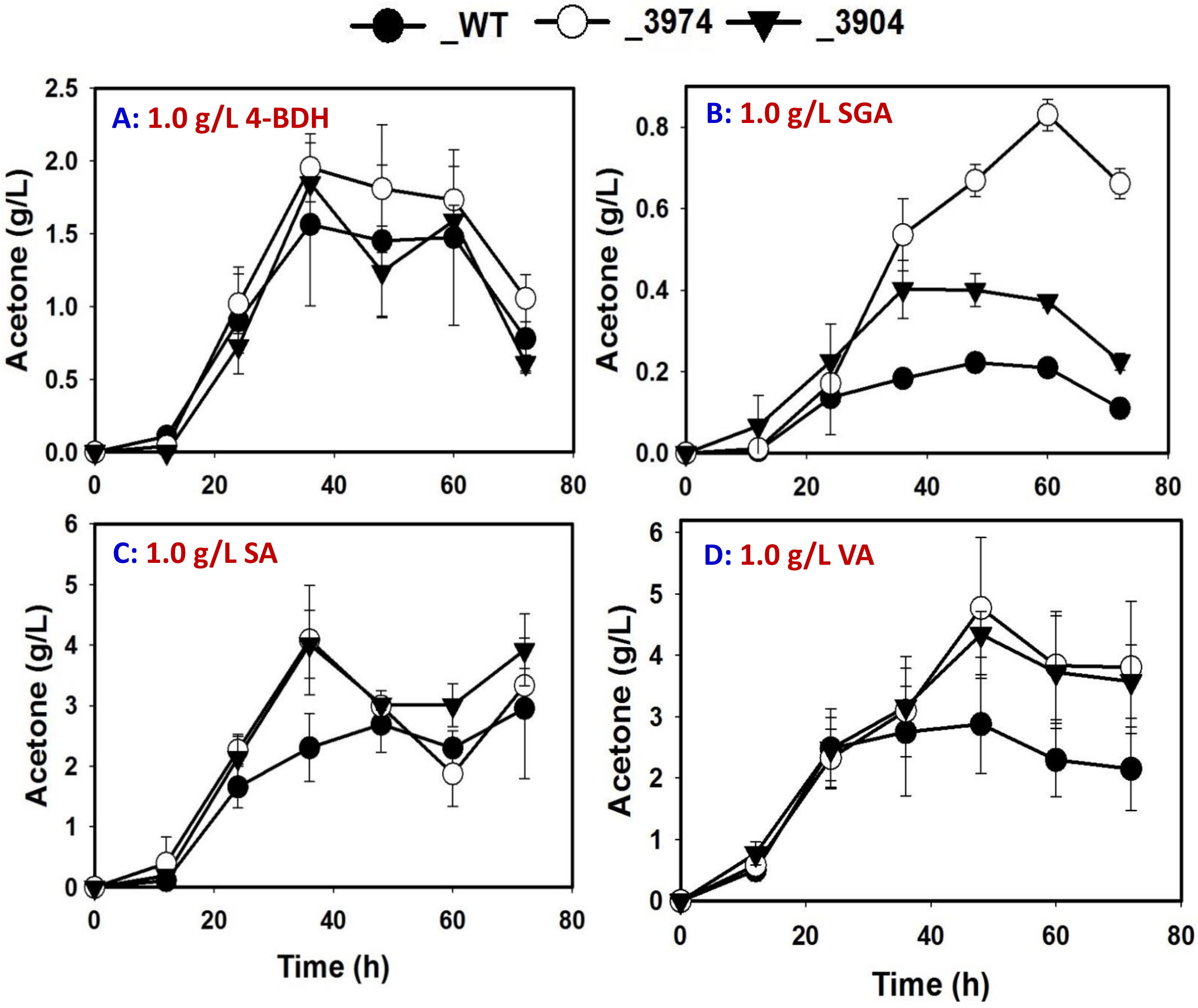

Figure S13. Acetone profiles of *Cb*_3974, *Cb*_3904, and *Cb*_wild type during fermentation in cultures supplemented with 0.75 g/L 4-hydroxybenzaldehyde (4-HBD), syringaldehyde (SGA), syringic acid (SA), and vanillic acid (VA) each.

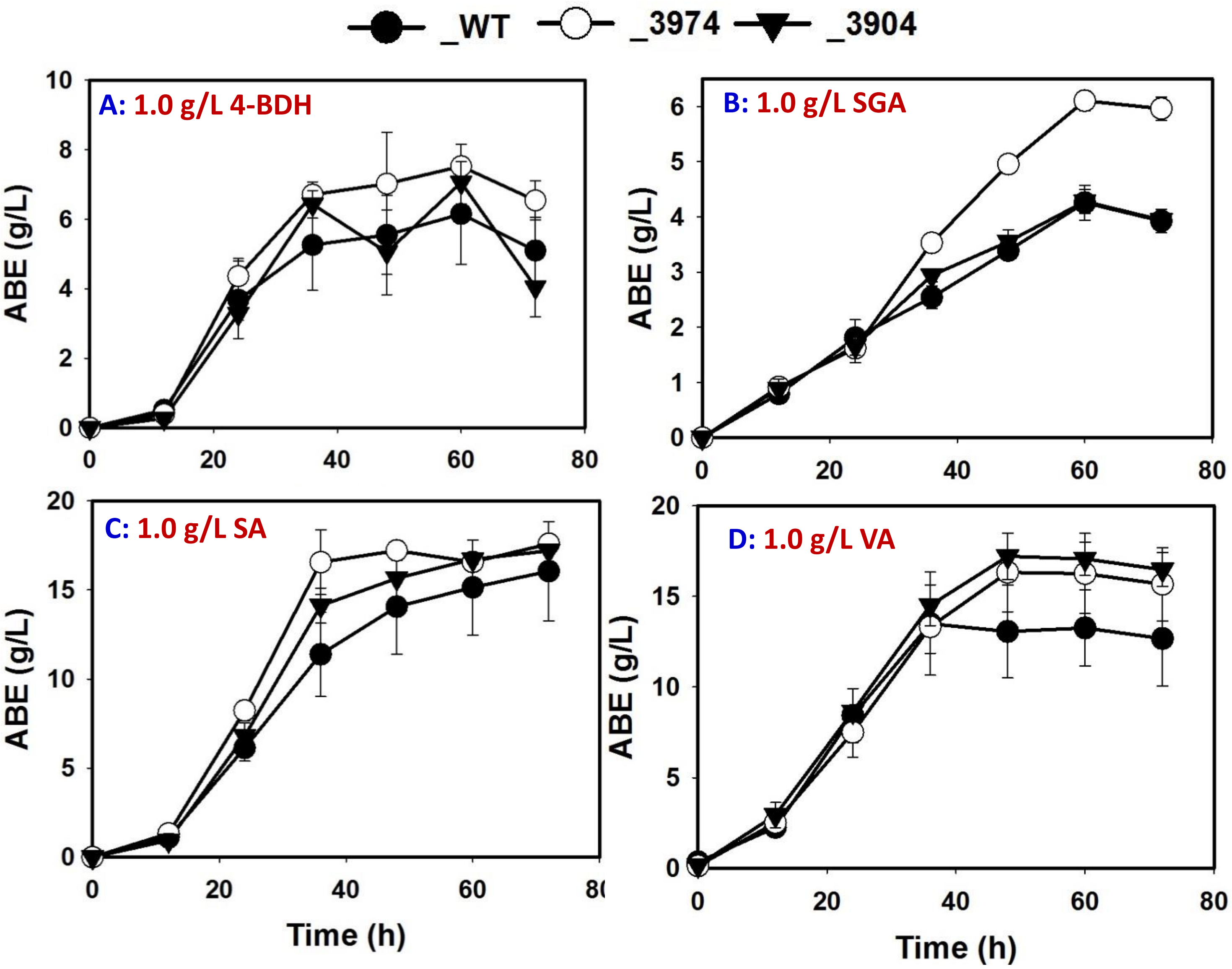

Figure S14. ABE profiles of *Cb*_3974, *Cb*_3904, and *Cb*_wild type during fermentation in cultures supplemented with 1.0 g/L 4-hydroxybenzaldehyde (4-HBD), syringaldehyde (SGA), syringic acid (SA), and vanillic acid (VA) each.

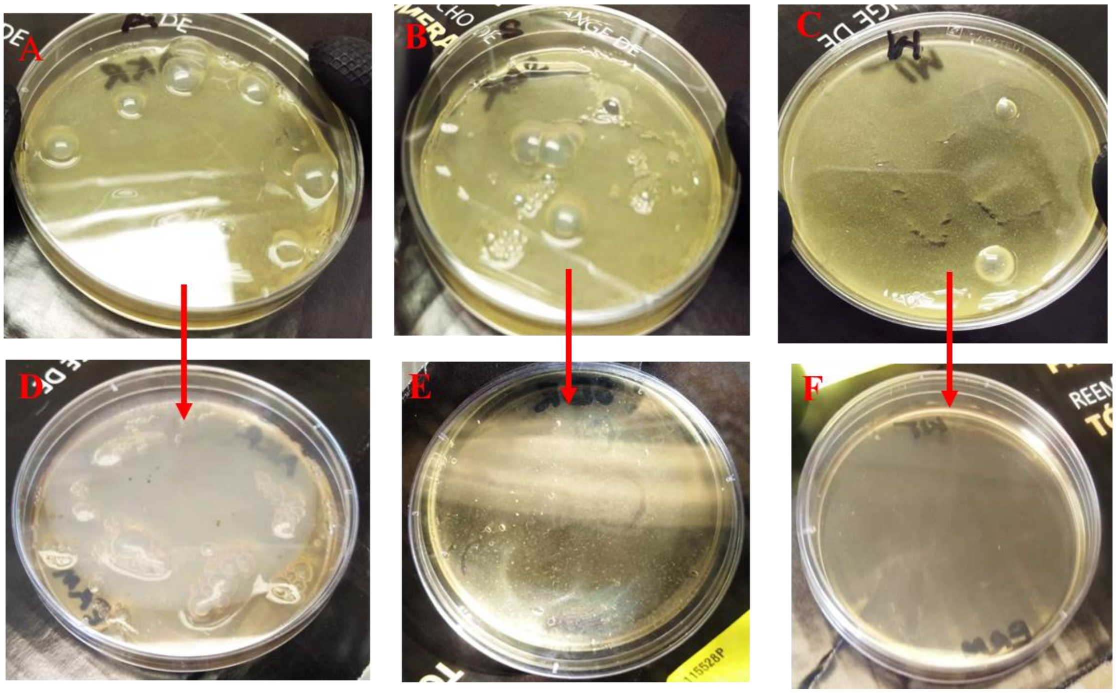

Figure S15. Replica plating of plasmid-cured *Cb*_3974 and *Cb*_3904. After plasmid-curing *Cb*_3974 and *Cb*_3904 were plated on TGY agar without erythromycin supplementation alongside *Cb*_wild type (A, B, C, respectively). Single colonies from *Cb*_3974 (**A**), *Cb*_3904 (**B**) and *Cb*_wild type (**C**) were picked and re-plated on fresh TGY agar supplemented with 25 µg/ml erythromycin. Colonies were observed in *Cb*_3974 (**D**) and *Cb*_3904 (**E**) and none in *Cb*_wild type (**F**). The red arrows indicate transfer of single colonies.

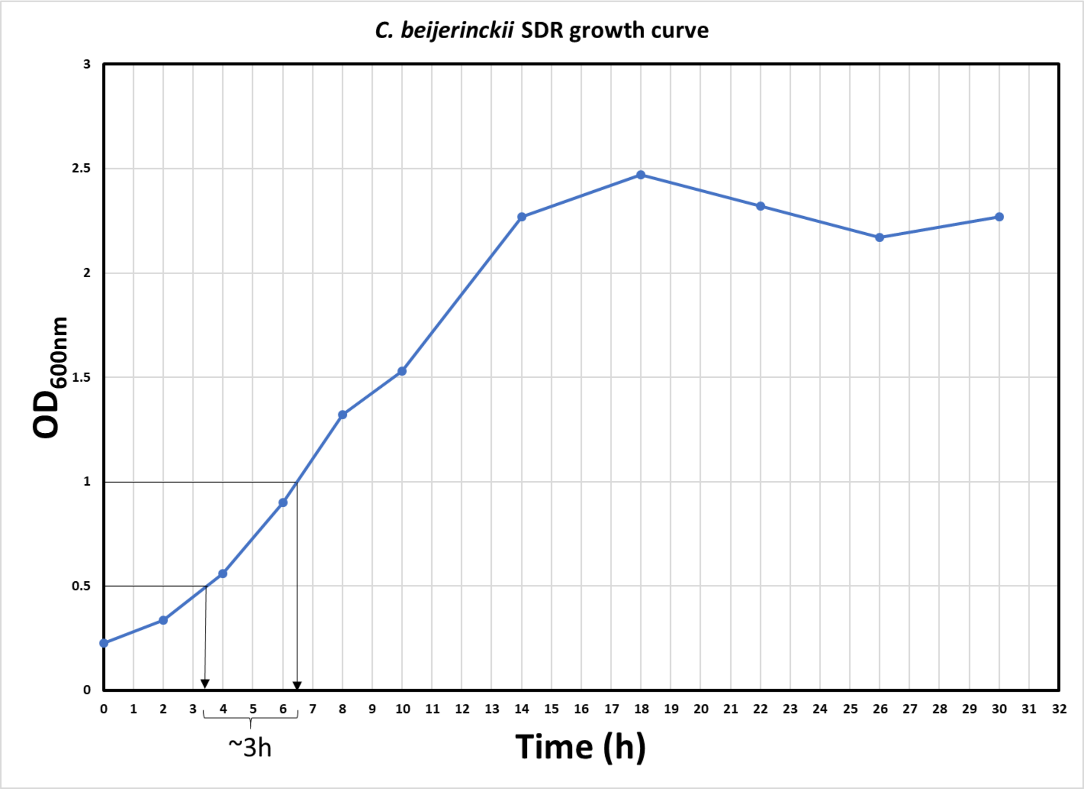

Figure S16. Determination of *Cb*_3904 generation time.

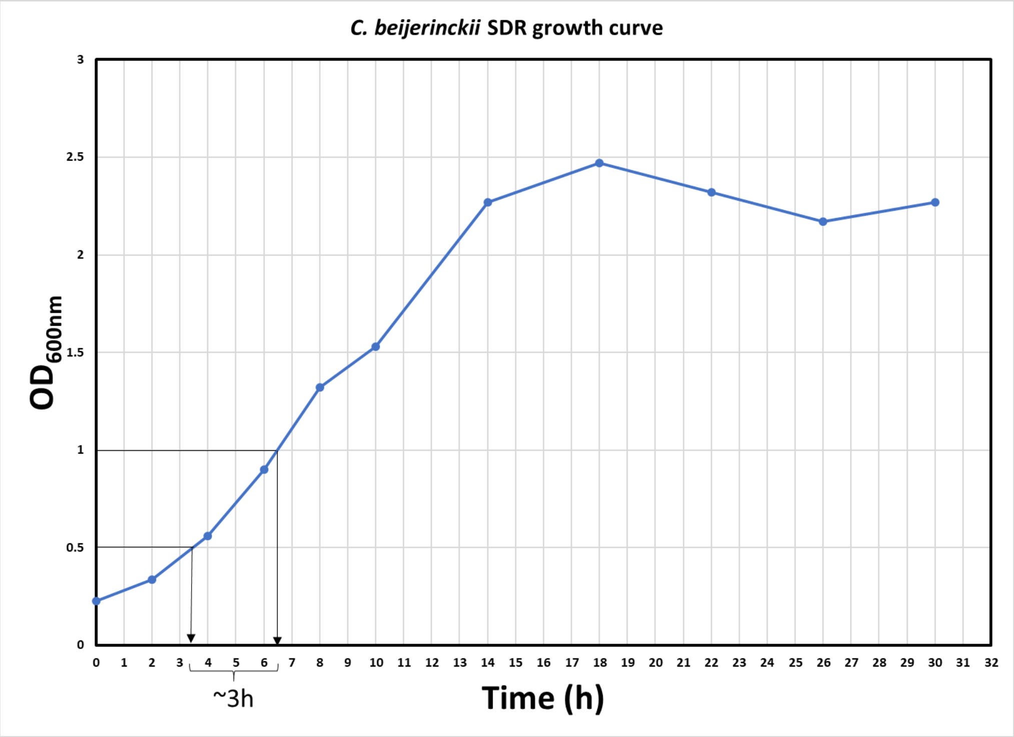

Figure S17. Determination of *Cb*_3974 generation time.

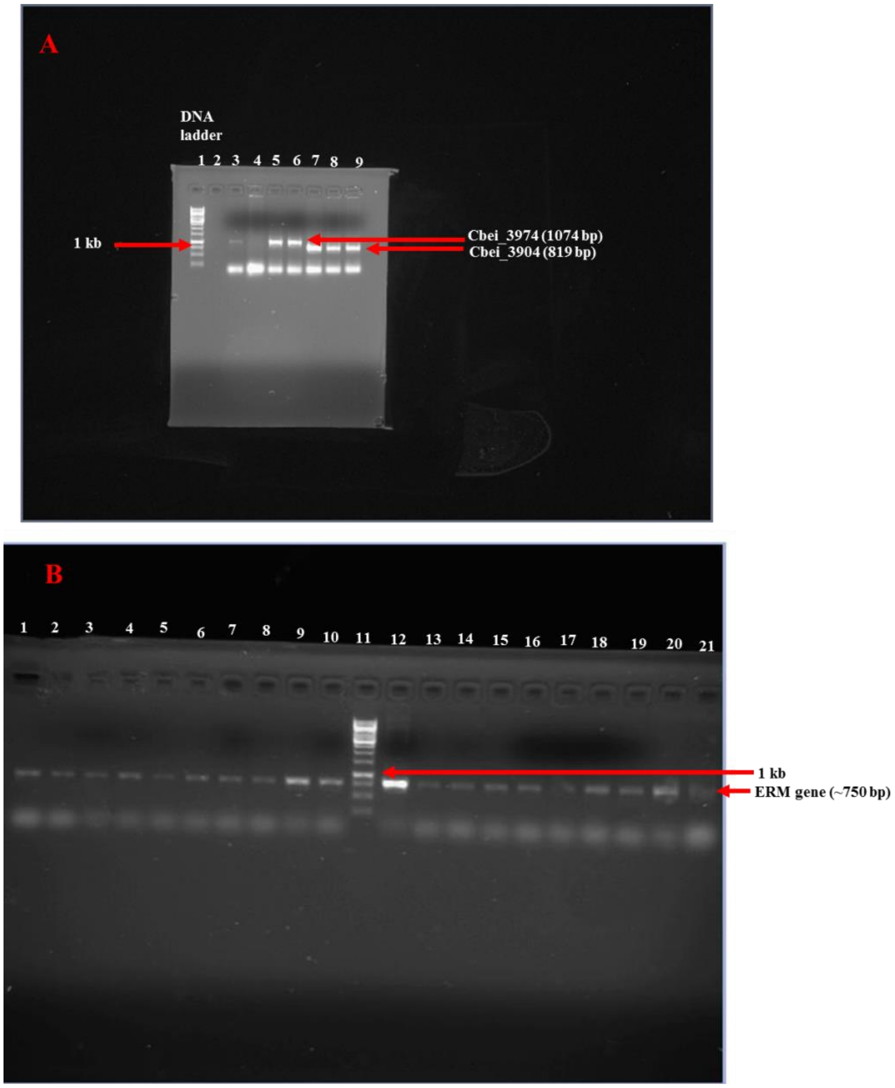

Figure S18. **Figure 12**. PCR amplification of respective constructs following extraction of recombinant plasmids from respective *E. coli* TOP 10 transformants. (**A**) *AKR* (*Cbei*_3974) and *SDR* (*Cbei*_3904) amplicons following PCR amplification with pMTL-JH16_3974 (lanes 4, 5 and 6) and pMTL-JH16_3904 (lanes 8 and 9) extracted from *E. coli* TOP 10 transformants as template. Lanes 3 and 7: positive controls, where lane 3 is the PCR amplicon using pMTL-JH16_3974 before transformation (into *E. coli*) as template, while lane 7 is the PCR amplicon using pMTL-JH16_3904 before *E. coli* transformation as template. (**B**) Gel image showing PCR amplicons of erythromycin resistance gene using genomic DNA extracted from *Cb* colonies transformed with pMTL-JH16_3904 (lanes 1-10) and pMTL-JH16_3974 (13-21). Lane 12: erythromycin resistance gene positive control. Lane 11: DNA ladder.

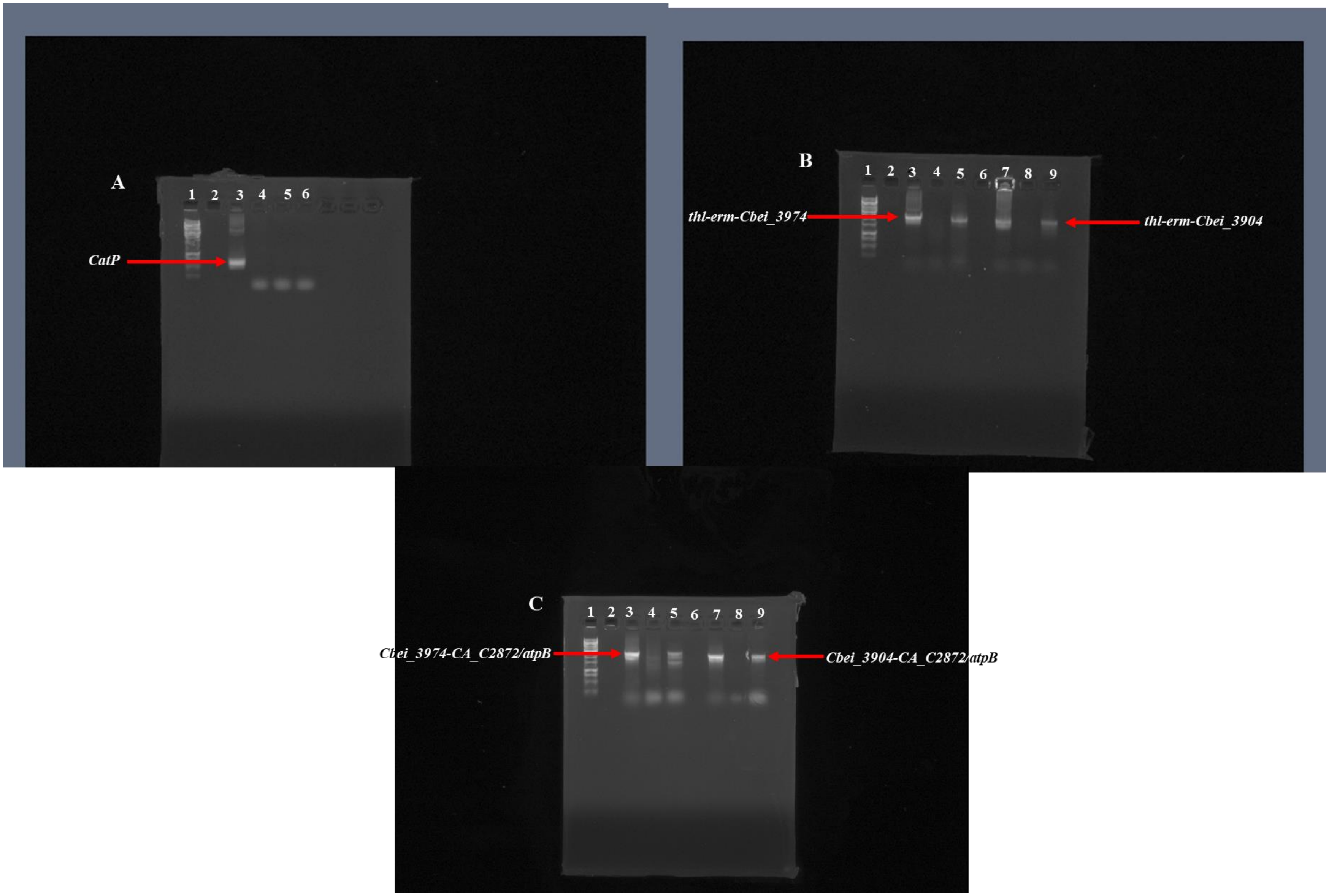

A
1 2 3 4 5 6
CatP
B
1 2 3 4 5 6 7 8 9
thl-erm-Cbei_3974
thl-erm-Cbei_3904
C
1 2 3 4 5 6 7 8 9
Cbei_3974-CA_C2872/atpB
Cbei_3904-CA_C2872/atpB

Figure S19. **Figure 2**. PCR amplification of the flanking regions of *Cbei_3974* and *Cbei_3904* using gDNA from plasmid-cured *Cb*_3974 and *Cb*_3904 as templates. (**A**) Gel image showing *CatP* amplicon following PCR with *CatP*-gene specific primers and the parent plasmid as template. Lane 1 is 1kb DNA ladder, lanes 3,4,5 and 6 are pMTL-JH16 (parent plasmid), and gDNA from *Cb*_wildtype, *Cb*_3974 and *Cb*_3904, respectively; (**B**) Gel image of PCR amplification of *thl*-*erm*-*Cbei*_3974 (or *Cbei*_3904) from respective gDNA samples. DNA ladder (lane 1), pMTL-JH16_3974 (lane 3), *Cb*_wildtype (lane 4), *Cb*_3974 (lane 5), pMTL-JH16_3904 (lane 7), *Cb*_wild type (lane 8), *Cb*_3904 (lane 9); (**C**) Gel image of PCR amplification of *Cbei*_3974 (or *Cbei*_3904)-*CA_C*2872/*atpB* from gDNA. DNA ladder (lane 1), pMTL-JH16_3974 (lane 3), *Cb*_wild type (lane 4), *Cb*_3974 (lane 5), pMTL-JH16_3904 (lane 7), *Cb*_wild type (lane 8), *Cb*_3904 (lane 9).
